# Adolescent athletes have better general than sports nutrition knowledge and lack awareness of supplement recommendations: a systematic literature review

**DOI:** 10.1017/S0007114523002799

**Published:** 2024-04-28

**Authors:** Susan C. Hulland, Gina L. Trakman, Rebekah D. Alcock

**Affiliations:** 1 Department of Dietetics, Nutrition and Sport, La Trobe University, Melbourne, VIC, Australia; 2 Essendon Football Club, Fitzroy, VIC, Australia

**Keywords:** Adolescent, Athletes, Nutrition knowledge

## Abstract

Nutrition knowledge (NK) impacts food choices and may be improved through educational programmes. Identifying knowledge gaps related to NK among adolescent athletes may guide future nutrition education programmes. This review aimed to systematically review the level of NK in adolescent athletes based on the currently available published literature. The protocol for this review was registered with PROSPERO (CRD42022321765). A literature search was conducted in April 2022 using MEDLINE, CINAHL, SPORTDiscus, Web of Science and SCOPUS databases. The study design was not restricted, provided that a quantitative NK score was reported for adolescent athletes. Studies were limited to the English language and published between 2010 and April 2022. Studies were assessed for quality and risk of bias using the Academy of Nutrition and Dietetics Quality Appraisal Checklist. Data extracted included demographics, questionnaire name, number of items, validation status and mean total and subsection NK scores. Meta-analyses were inappropriate due to the heterogeneity of NK assessment tools; therefore, results were presented narratively. Thirty-two studies that assessed NK of 4553 adolescent athletes and 574 comparison participants were included. Critical appraisal of studies resulted in neutral rating ‘moderate quality’ for most (*n* 30) studies. Studies lacked justification for sample size and often used inadequately validated questionnaires. NK scores ranged from poor (33·3 %) to excellent (90·6 %). The level of NK across studies is difficult to determine due to heterogenous questionnaires often lacking appropriate validation. NK should be assessed using tools validated in the relevant population or revalidated tools previously used for other populations.

Athletes’ nutrition choices influence sporting performance and recovery post-exercise. Carefully timed pre-, during and post-training foods and fluids ensure adequate energy availability during exercise and training, aid glycogen re-synthesis post-exercise and maximise training adaptations^(1,2)^.

The nutritional choices of adolescent athletes are of heightened significance as they face a period of rapid growth and development, during which nutrient intake may influence neurodevelopment, bone mineral density and the risk of chronic illnesses^(3,4)^. Adhering to population-based dietary guidelines and sport-specific dietary recommendations can provide long-term health benefits and athletic advantages^(1,5,6)^. Despite the benefits of following dietary recommendations, a study of Australian adolescent rugby players found that players were consuming inadequate vegetable serves, excessive energy was derived from discretionary choices (foods typically high in saturated fat/sodium and/or low in fibre and micronutrients) and carbohydrate intake was insufficient when compared with the Sports Dietitians Australia recommendations^(7)^. Similarly, low to moderate adherence to recommended dietary guidelines has been observed in adolescent Spanish beach handball players^(8)^, adolescent Cypriot swimmers^(9)^ and adolescent Brazilian volleyball players^(10)^. Considering the implications of non-adherence to both population-based and sport-specific dietary recommendations for short- and long-term health and athletic performance, it is pertinent to investigate the factors influencing adolescent athletes’ food choices.

Birkenhead and Slater’s^(11)^ theoretical framework describes the influence of multiple factors on athletes’ food choices. Trakman^(12)^ proposed that these factors may be categorised as modifiable, semi-modifiable, or non-modifiable based on their potential to change when professional guidance and education are provided. No factor can be considered solely responsible for athletes’ food choices; rather, these factors are dynamic, and the level of influence may be situational. Of particular significance is the factor of nutrition knowledge (NK); the level of NK can not only inform nutrition choices but may also be measured with relative ease and compared using quantitative NK assessment where a numerical score is provided to reflect NK. Furthermore, the introduction of educational intervention programs can result in measurable improvement in this area, as reported in previous systematic literature reviews of athletes’ NK^(13,14)^.

The success of a nutrition education programme relies first on an understanding of areas of importance to the targeted population, and second on identifying common knowledge gaps within the population^(15)^. In this context, several researchers have evaluated NK in adolescent athletes using a cross-sectional research design.

Previous systematic literature reviews have reported the NK of children and adolescents^(16)^, the NK of solely adult athletes^(14,17)^ or both adolescent and adult athletes^(18)^. Thakur and Mathur^(16)^ reviewed studies investigating the relationship between NK and dietary behaviour among children and adolescents and found that higher NK was significantly associated with underweight or normal weight based on BMI, indicating that NK may influence dietary choices. However, only two of the included studies referred to physical activity, with no description of athletic ability or comparison between physically active and inactive adolescents. Therefore, the results of this review cannot be generalized to adolescent athletic populations. A 2016 review by Trakman *et al*.^(14)^, which was subsequently updated by Janiczak *et al*.^(17)^ in 2021, excluded adolescent athletes and noted that NK was generally poor, and that several studies reported a statistically significant relationship between age and NK. Nevertheless, excluding adolescent athletes in the review leaves the conclusions unlikely to be transferrable to the adolescent athlete population^(19,20)^. Heaney *et al*.^(18)^ included both adult and adolescent athletes over 13 years of age in their systematic literature review and concluded that the heterogeneity of NK assessment tools used, lack of validation methods, and unclear demographic descriptions prevented clear conclusions from being drawn on the NK level of athletes and their non-athletic comparators. Since the review by Heaney *et al*.^(18)^, a plethora of research concerning adolescent athletes’ NK levels has been conducted^(9,19–49)^, presenting the need to evaluate the currently available research to determine the level of NK in adolescent athletes. This review will adapt the protocol of the previous review^(18)^ to target an adolescent athlete population.

## Objectives

Considering the role of nutrition in athletic performance and NK as a modifiable factor influencing food choice, and gaps in existing reviews, this review aims to:

1. Assess the level of general nutrition knowledge (GNK) and sports nutrition knowledge (SNK) among adolescent athletes between 10 and 19 years of age.
2. Identify common areas of misunderstanding within subtopics of NK, including GNK and SNK, and hydration.
3. Comparison of NK levels among adolescent athletes and non-adolescent athlete groups, including coaches, parents, adult athletes and adolescent non-athletes.

## Methods

This systematic literature review was conducted following the PRISMA guidelines^(50)^ (Supplementary Material – Table S1). The protocol was registered with PROSPERO (protocol registration ID CRD42022321765) and may be retrieved from https://www.crd.york.ac./prospero/display_record.php?RecordIDuk=321765.

### Eligibility criteria

The WHO’s definition of an adolescent (10–19 years of age) was employed^(51)^. Where the age range extended beyond 10–19 years, articles were included if the mean age was between 10·0 years–19·0 years. Athletes were defined as individuals who participated in any organised sport competitively, including aesthetic sports such as gymnastics, dancing and figure skating. All levels of sports were included; for example, recreational, competitive, high school and elite.

The articles included in this review were original studies (cross-sectional, observational and randomised/non-randomised controlled trials, intervention), which provided a quantitative NK score in an adolescent athlete population. Studies reporting general nutrition, sports nutrition or related domains, such as hydration and supplements, were included. Only baseline data were extracted if more than one NK assessment was conducted. NK needed to be converted into a % of correct answers and reported separately from any other assessed domain, such as attitude towards nutrition. Where studies stratified NK scores into multiple groups other than gender and sport played, the review author calculated a pooled result for ease of comparison.

Studies with or without comparison groups were included. The comparison groups included parents, coaches, adult athletes and nonathletes of any age.

The method of questionnaire administration (e.g., handwritten, electronic, self-administered and interviewer-administered) and setting (e.g., online, at school and athletic training) were not restricted.

### Exclusion criteria

The year of publication was restricted to 2010 onwards, as a comparable systematic review assessed the NK of athletes, including adolescents, for studies conducted before 2010^(18)^. Articles not published in English were excluded. Grey literature, abstracts, conference posters, editorials and unpublished theses were excluded. Qualitative studies were excluded. Table 1 presents the inclusion and exclusion eligibility criteria of this review.

**Table 1.** Eligibility criteria

Included	Excluded
Original research, study designs included: cross-sectional, randomised/non-randomised controlled trial, observational, intervention	Abstracts, conference posters, editorials, letters to the editor, literature reviews, and unpublished theses
Adolescent athletes (10–19 years) of any sport type and level	Mean age beyond the range of 10–19, all non-athletes
English language	Non-English language
Quantitative nutrition knowledge score including GNK, SNK, and related sub-domains	Qualitative studies, or studies that did not provide quantitative NK score
Published between 2010 and April 2022	Published before 2010

### Information sources and search strategy

Searches were conducted in April 2022 by Susan Hulland (SH) using the following databases: MEDLINE, SportDiscus, SCOPUS, Web of Science and CINAHL. In the MEDLINE search, ‘adolescent’, ‘athlete’ and ‘nutrition knowledge’ were mapped to subject headings: ADOLESCENT, ATHLETE and NUTRITIONAL SCIENCES, respectively. Keywords were then added to the search: adolescen* OR junior OR youth OR teen* OR child* OR ‘young adult’ AND athlet* OR sport* AND ‘nutrition* knowledge’ OR ‘nutrition* questionnaire’ OR ‘nutrition* awareness’. The syntax of these terms was appropriately adjusted for each database (online Supplementary Material – Table S2). The search was limited to the English language. The year of publication was not limited during the search, as articles published before 2010 were manually excluded during the screening process. This was done to assess the potential publication bias of this review by identifying articles that would otherwise be included if not for the publication date. The reference lists of the included articles were screened to identify any additional eligible articles. Where possible, ‘saved search alerts’ were applied to retrieve articles published between the time the search was conducted, and the systematic literature review was completed in May 2022.

### Study selection

Studies retrieved from the database search were exported to Endnote for duplicate removal and subsequently exported to Covidence for eligibility screening. Two reviewers independently conducted title and abstract screening for eligibility (SH and Gina Trakman (GT) or Rebekah Alcock (RA)), with a third reviewer resolving conflicts (GT or RA). Studies that met the inclusion criteria were then screened in full text by two reviewers (SH and GT), and conflicts were resolved by discussion between the two reviewers to reach a consensus. A third reviewer (RA) resolved outstanding conflicts if consensus could not be achieved through discussion.

### Data collection process

A purpose-designed data extraction form based on the Academy of Nutrition and Dietetics Data Extraction Template^(52)^ was used to extract data from the included studies. Two reviewers (SH and RA) independently extracted data from a sample (*n* 5) of the included articles and reached a consensus of >90 %; discrepancies were resolved by discussion and SH conducted the remainder of the data extraction. A third reviewer (GT) checked the data extracted for inconsistencies. For each of the included articles, extracted data included author, year of publication, study information (location, study design, aim, funding sources and inclusion and exclusion criteria), demographic information of participants and comparison group where applicable (number, age, gender, type of sport played and level of sport), questionnaire information (questionnaire name and/or author, number of questions and subsections) and outcome measures (total % score, subsection % scores where reported).

Validation of the questionnaire, as described by the author, was defined in the following categories: yes, partial, no and unclear. Studies were rated as ‘yes’ if the authors validated the tool they used OR if they used a previously validated tool, including if any minor modifications were undertaken, i.e. re-worded to apply to the local population, for example, kilojoules replacing calories. Studies were rated as ‘partial’ if they modified a previously validated questionnaire, but it was unclear if modifications were ‘minor’ or if the authors did not describe attempts to re-validate the questionnaire after modification. Studies were rated as ‘no’ if the authors used an unvalidated questionnaire or made major modifications to a previously validated questionnaire without revalidation. An ‘unclear’ rating was given if the authors did not describe validation, and it was not possible to retrieve the original article to assess validation.

In cases where data were missing or there appeared to be inconsistencies, the original author was contacted for clarification and/or raw data. For inclusion in this review, the author was given a period of 14 d to respond to a request for further information.

### Quality appraisal/risk of bias in individual studies

The quality of each included article was appraised using the Academy of Nutrition and Dietetics “Quality Criteria Checklist for Primary Research”^(52)^. Two reviewers (SH and RA) independently appraised a sample of articles (*n* 5) that reached a consensus on >90 % of the criteria. The remaining articles were appraised by a single reviewer (SH). This quality appraisal checklist was selected for use because it can be applied to all study designs included in this review, ranking an article as ‘positive’, ‘negative’ or ‘neutral’. The checklist assesses each article based on four criteria regarding relevance and ten validity criteria that are individually awarded ‘yes’, ‘no’, ‘unclear’ ‘n/a’. A positive rating for the overall quality appraisal was determined by receiving ‘yes’ for the majority of criteria, and specifically ‘yes’ for criteria 2, 3, 6 and 7 were required when applicable.

Criteria 2 addresses selection bias in study selection. To receive a positive rating, articles were required to describe the demographic characteristics, including age, gender and type of sport, along with a justification for the sample size used and a description of the broader relevant population. Criteria 3 was not relevant for most studies as it addresses whether study groups were comparable, as such cross-sectional studies and some intervention studies received ‘n/a’ for criteria 3. Receiving ‘n/a’ for this item did not hinder the potential for an overall positive rating. Criteria 6 assesses whether the intervention or procedure was discussed in detail. For this review, articles were marked as positive if they provided details of the study procedure, including ethics approval, consent obtained and the method of administering the questionnaire. Criteria 7 refers to the validity and reliability of the measurements used, as such articles received a ‘yes’ when they reported using a validated questionnaire or conducted measures to re-validate after modifying an existing questionnaire.

### Summary measures

The principle outcome measure was the quantitative NK score. NK scores were retrieved as the mean correct NK knowledge score and mean correct scores for all reported subsections relating to NK, including GNK, SNK, hydration, supplements, and any other reported subsection relating to NK. All mean scores were converted to percentages to maintain consistency when discussing results. Comparisons were conducted by comparing the differences in mean scores. The secondary outcomes of identifying knowledge gaps relating to nutrition and differences between participants and comparison groups were also conducted by comparing the difference in mean scores.

### Synthesis of results

Owing to the heterogeneity of the NK assessment tools used in the included articles, a meta-analysis and statistical measures of comparison could not be performed. As such, the results of this review will be described narratively based on the NK assessment tool used, NK domain assessed (GNK, SNK, hydration or supplements), comparisons within studies, including the presence of a non-adolescent athlete comparison group, and the relationship between NK and age.

A narrative synthesis will also be provided regarding the quality of the included studies, focusing on criteria 2, 6, and 7 of the Academy of Nutrition and Dietetics ‘Quality Criteria Checklist for Primary Research’^(52)^ as the primary criteria determining the quality rating.

### Risk of bias across studies

Publication bias of this review due to articles not captured within the inclusion criteria was explored based on the age of participants, date of publication and language of included articles and will be discussed qualitatively. Reporting bias was addressed by contacting authors when data were missing, or inconsistencies appeared in the reported data.

## Results

### Study selection

**Fig. 1.**
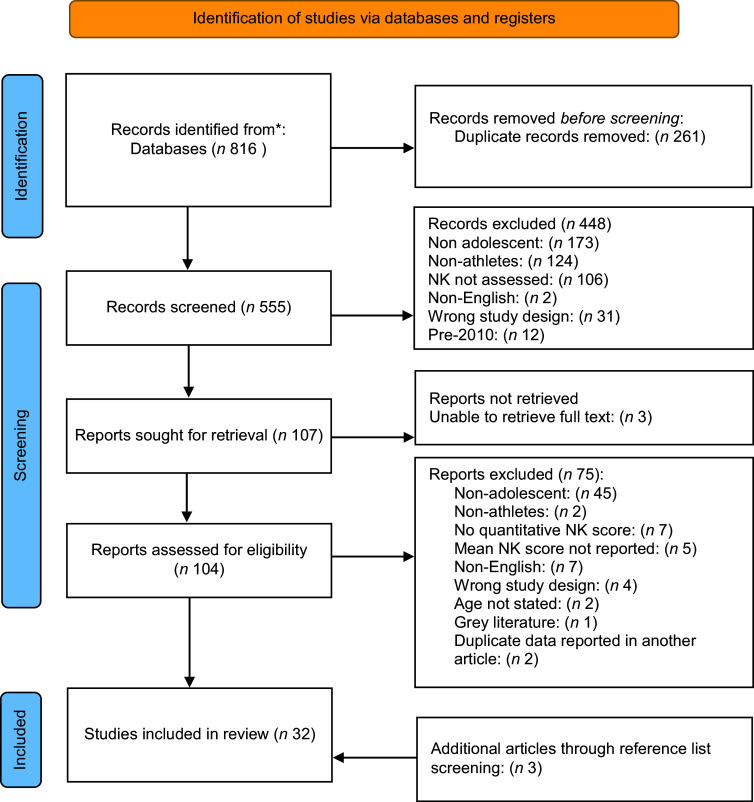
PRISMA flow diagram of the inclusion eligibility screening process.

The search of databases resulted in 816 articles, after the removal of duplicates (*n* 261) a total of 555 articles were included for the title and abstract screening. Two hundred and sixteen articles were excluded during the title and abstract screening, including eleven articles that were published before 2010. A total of 107 articles were considered eligible for full-text screening, with three articles unable to be retrieved in full text through the university document delivery portal or via contact with the study authors^(48,53,54)^. As such, 104 full texts were screened, with thirty-one articles eligible for inclusion in the review. The reasons for the exclusion of articles during screening are detailed in the supplementary material (online Supplementary Table S3). Screening of the reference lists provided an additional three articles for review. During data extraction, two longitudinal intervention studies^(55,56)^ were excluded to avoid duplicate publication bias, as their baseline data were reported in standalone articles already included in the review (Fig. 1).

### Study characteristics

Table 2 provides the results of the individual studies. Most of the studies (*n* 22) used a cross-sectional design. Of the remaining studies, nine investigated the impact of nutrition education in before–after studies (randomized controlled trial (*n* 3), quasi-experimental trial (*n* 6)) and one crossover trial regarding hydration status and sporting performance. Eighteen countries were represented across five continents: Europe (*n* 15), South America (*n* 5), North America (*n* 5), Asia (*n* 5) and Australia (*n* 2). African countries were not represented in any of the included studies.

**Table 2.** Summary of results

Author, year, country	Study design	Sport played; *sport level*	Adolescent athletes (*n*, gender)	Adolescent athlete’s age (mean or range if mean NR)	sd	Non-adolescent athlete comparison (*n*, gender)	Non-adolescent athlete comparison group age (mean or range if mean NR)	sd	Questionnaire used, number of questions	Validation
Aguilo *et al*.^(21)^, (2021): Spain	Quasi-experimental	Gymnastics; *competitive*	*n* 24, F:24	14·1	2·3	n/a	n/a		Adapted from GNKQ Parmenter and Wardle^(60)^, (1999); *n* 61	Partial
Altavilla *et al*.^(22)^, (2017): Spain	Cross-sectional	Swimming; *competitive*	*n* 86, F:46 M:40	11–16		n/a	n/a		Adapted from Zawila *et al*.^(62)^, (2003); *n* = NR	Unclear
Argolo *et al*.^(23)^, (2018); Brazil	Cross-sectional	Table tennis; *competitive*	*n* 25, F:2 M:23	*n* 13·8	2·5	*Adult athletes: n* 17 M:17	33·6	10·8	Leite *et al*.^(61)^ (2016); *n* 14	Yes
Atkins *et al*.^(24)^, (2021); USA	Non-randomised controlled trial	American football; *high school*	Group 1: *n 21, M:21*	Group 1:*16*	*11*	n/a	n/a		Modified Decher *et al.* ^(70)^, (2008); *n* 25	No
			Group 2*: n* 20, M:20	Group 2:16						
Bakhtiar *et al*.^(25)^, (2021); Bangladesh	Cross-sectional	Cricket, football, athletics, basketball, volleyball, archery, table tennis, karate, wushu, taekwondo, hockey;	*n* 260, F:27 M:233	15·5	1·83	n/a	n/a		Author designed; *n* 22	Yes
		*Level NR*								
Bird and Rushton^(26)^, (2020); Australia	Cross-sectional	Netball, officiating, basketball, softball, hockey, tennis, lawn bowls and triathlon;	*n* 101, F: 64 M: 37	15·3	1·4	n/a	n/a		Zinn *et al*.^(59)^, (2005); *n* 90	Yes
		*Competitive*								
Calella *et al*.^(27)^, (2021); Italy	Cross-sectional	Gym members, volleyball, swimmer, gymnasts; *regional /national competitors*	Total *n* 131, *Volleyball: n* 43, F:21, M:22 *Swimmers: n* 39, F:19, M: 20 *Gymnasts: n* 49, F:49	*Volleyball:* 17·7 *Swimmers:* 16·6	1·5	Total *n* 80, *Gym members: n* 33, F:16 M:17, *Inactive youth: n* 47, F:28 M: 19	*Gym members:* 16·7 *Inactive youth:* 16·7	1·1	GeSNK, Calella *et al.* ^(71)^, (2017); *n* 62	Yes
				*Gymnasts:* 16·2	1·8			1·5		
					1·8					
Carvalho *et al*.^(28)^, (2011); Portugal	Cross-over trial	Basketball; *national competitors*	*n* 12, M:12	14·8	0·45	n/a	n/a		Modified Nichols *et al*.^(63)^, (2005); *n* 16 hydration	Partial
Chia *et al*.^(19)^, (2015); Singapore	Cross-sectional	NR; *high school*	*n* 586, F: 259 M: 322 Other:5	13·9	2·5	n/a	n/a		Author designed; *n* 30	Yes
Daniel *et al.* ^(29)^, (2016); Brazil	Intervention	Volleyball; *state competitors*	*n* 10, F:10	17·2	0·94	n/a	n/a		Jurgensen *et al*.^(72)^, (2015); *n* = NR	Yes
Escribano-Ott *et al*.^(30)^ (2021); Spain	Cross-sectional	Basketball; *competitive*	*n* 69, F:37 M:32	15–18		*Adult athletes*	*Non-pro: NR*		Zinn *et al*.^(59)^, (2005); *n* 23	Partial
						*Non-pro: n 14, F:8 M:6 Pro: n* 21, F:10 M:11	*Pro: NR*			
Foo *et al*.^(31)^, (2021); UK	Quasi-experimental	Swimming; *competitive*	*n* 15, F:10 M:5	15·5	1·1	n/a	n/a		Walsh *et al*.^(57)^, (2011); *n* 16	No
Gonçalves *et al*.^(32)^, (2014); Brazil	Intervention	NR; *competitive*	*n* 58, F:27 M:31	13·7	0·77	n/a	n/a		Modified Lima *et al*.^(73)^, (1985). Triches & Giugliani^(74)^, (2005); *n* = NR	Unclear
Hardy *et al.* ^(33)^, (2017); USA	Cross-sectional	NR; *College*	*n* 95, NR	18–19		*n* 99, NR	>20		Modified GNKQ, Parmenter and Wardle^(60)^, (1999); *n* = NR	Partial
Heikkilä *et al*.^(35)^, (2018); Finland	Cross-sectional	Cross-country skiing, orienteering, biathlon, running/racewalking, triathlon, swimming/rowing/canoeing, athletics, cycling; *competitive*	*n* 312, F:156 M:156	17·9	1·2	*Coaches: n* 94, F:25 M:69	44·3	12·3	Heikkilä *et al*.^(58)^, (2018); *n* 79	Yes
Heikkilä *et al.* ^(34)^, (2019); Finland	Randomised controlled trial	Cross-country skiing, orienteering, biathlon, running/race walking, triathlon; *competitive*	Group 1: *n* 37, F:18 M:19 Group 2: *n* 42, F:17 M:25	Group 1:18·0	1·4	n/a	n/a		Heikkilä *et al*.^(58)^, (2018); *n* 78	Yes
				Group 2:18·0	1·4					
Jusoh^(36)^, (2014); Malaysia	Cross-sectional	Football, athletics, Sepak takraw, hockey, rugby, netball; *regional/state/national competitors*	*n* 70, F:25 M:45	14·0	1	n/a	n/a		Modified Nichols *et al.* ^(63)^, (2005); *n* 10	Partial
Jusoh *et al*.^(37)^, (2021); Malaysia	Cross-sectional	Handball; *national competitors*	*n* 312, F: 157 M:155	16	1	n/a	n/a		Modified Razalee and Tan^(75)^, (2014); *n* 11	Yes
Kettunen *et al.* ^(38)^ (2021); Finland	Cross-sectional	Cross-country skiers; *national competitors*	*n* 19, F:19	16·7	0·7	n/a	n/a		Heikkilä *et al*.^(58)^, (2018); *n* 79	Yes
Laramée *et al*.^(39)^ (2017); Canada	Randomised controlled trial	Various aesthetic sports: synchronised swimming, gymnastics, dancing, cheerleading; *competitive*	Group 1: *n* 37, F:37	Group 1:14·1	1·5	n/a	n/a		Morisette *et al*.^(76)^, (2015); *n* 37	Yes
			Group 2: *n* 33, F:33	Group 2:13·1	1·2					
Mandic *et al*.^(40)^, (2013); Croatia/Serbia	Cross-sectional	Synchronised swimming; *competitive*	*n* 82, NR	17	1·92	*Coaches:*	30	5·26	Kondric *et al*.^(77)^, (2013); *n* 18	Yes
						*n* 28, NR				
Manore *et al*.^(41)^, (2017); USA	Cross-sectional	Soccer; *high school*	*n* 535, F:297 M:238	15·3	1·14	n/a	n/a		Walsh *et al.* ^(57)^, (2011); *n* 12	No
Nascimento *et al.* ^(42)^, (2016); Brazil	Quasi-experimental	Fighting, athletics, cycling, swimming, tennis, beach volleyball, surfing, rowing, sailing; *competitive*	*n* 21, F:6 M:15	15·4		*Adult athletes: n* 11, M:11	23·7		Leite *et al.* ^(61)^, (2016); *n* 14	Yes
Noronha *et al*.^(43)^, (2020); Brazil	Cross-sectional	Soccer; *state competitors*	*n* 73, M:73	17·0	1·3	n/a	n/a		Leite *et al*.^(61)^, (2016); *n* 14	Yes
Philippou *et al*.^(9)^, (2017); Cyprus	Intervention	Swimming; *competitive*	*n* 34, F:11 M:23	15·2	1·5	n/a	n/a		Author designed; *n* 10	Yes
Sanchez-Diaz *et al.* ^(44)^, (2021); Spain	Cross-sectional	Basketball; *elite*	*n* 23, F:10 M:13	F: 12·7 M:13·5	0·5	n/a	n/a		Modified Turconi *et al.* ^(78)^,	Yes
					0·3				(2003); *n* 11	
Saribay and Kirbay^(45)^, (2019); Turkey	Cross-sectional	Football, basketball, volleyball, athletics, handball, other; *high school/ college*	*n* 495, F:100 M:395	14–19		n/a	n/a		Öz *et al.* ^(79)^, (2016); *n* 38	Yes
Spendlove *et al*.^(46)^, (2012); Australia	Cross-sectional	Life-saving, rugby league, other (NR); *elite*	*n* 175, F:99 M:76	18·9	4·9	*Community* (CM): *n* 116, F:84 M:32	CM: 21·9	4·2	Modified GNKQ, Parmenter and Wardle^(60)^, (1999); *n* 45	Yes
						*Dietetic trained (DT): n* 53, F:46 M:7	DT: 29·1	8·5		
Supriya & Ramaswami^(47)^, (2013); India	Cross-sectional	Track and field; *state/national competitors*	*n* 178, F:107 M:71	18·0	3·2	n/a	n/a		Author designed; *n* 10	Partial
Walsh *et al.* ^(57)^, (2011); Ireland	Cross-sectional	Rugby; *high school*	*n* 203, M:203	16–18		n/a	n/a		Author designed; *n* 16	No
Webb and Beckford^(49)^, (2014); Trinidad and Tobago	Cross-sectional	Swimming; *competitive*	*n* 220, F:98 M:122	14·6	2·5	n/a	n/a		Adapted Ozdogan and Ozcelik^(80)^, (2011); Zawila *et al*.^(62)^, (2003); *n* 21	Partial
Wyon *et al*.^(20)^ (2014); UK	Cross-sectional	Ballet; *elite*	*n* 139, F:86 M:53	11–18		Professional ballet dancers *n* 41, F:25 M:16	19–39		GNKQ, Parmenter and Wardle^(60)^, (1999); NR	Partial

Author, year, country	Topic and subsections (if reported)	Adolescent athletes mean correct total (%)	sd	Adolescent athletes mean correct subsection (%)	sd	Comparison group Mean correct total (%)	sd	Comparison group Mean correct subsection (%)	sd	Quality rating
Aguilo *et al*.^(21)^, (2021): Spain	GNK	45·9	2·79^†^	NR		n/a		n/a		Neutral
Altavilla *et al*.^(22)^, (2017): Spain	SNK	33·3	12·6	Hydration: 28·6	28·6	n/a		n/a		Neutral
	Hydration^(4)^									
Argolo *et al*.^(23)^, (2018); Brazil	GNK^(3)^	55	12	GNK: 82·5	23*	66·7	10	GNK: 97	9·4*	Neutral
	Brazilian food pyramid (BFP)^(1)^			BFP: 14	8			BFP: 17·6	10	
	SNK^(10)^			SNK: 63·2	20			SNK: 79·4	21*	
Atkins *et al*.^(24)^, (2021); USA	GNK	Group 1:90	6	NR		n/a		n/a		Neutral
	Hydration	Group 2:89	9							
Bakhtiar *et al*.^(25)^, (2021); Bangladesh	GNK	58·0	9·9^†^	NR		n/a		n/a		Neutral
Bird and Rushton^(26)^, (2020); Australia	Nutrients, dietary reference intake (DRI), Recovery, weight gain, weight loss, supplements	43·8	11·4	Nutrients: 51, DRI: 27, hydration: 46, recovery: 54, Weight gain: 3–78, weight loss: 56, supplements: NR		n/a		n/a		Neutral
Calella *et al*.^(27)^, (2021); Italy	GNK^(29)^, SNK^(33)^	Total: Volleyball: 60·9 Swimmers: 62·4 Gymnasts: 66·6	12·3	GNK – Volleyball: 63·6 Swimmers: 63·0 Gymnasts: 68·4 SNK – Volleyball: 55·8 Swimmers: 63·0 Gymnasts: 63·0	13·8	Total: Gym members: 49·6 Inactive youth: 53·2	14·3	GNK – Gym members: 50·6 Inactive youth: 55·5 SNK – Gym members: 47·6 Inactive youth: 48·8	15·8	Neutral
			7·9		10·3		8·7		10·9	
			11·5		12·5				15·8	
					13·3				13·0	
					10·9					
					13·3					
Carvalho *et al*.^(28)^, (2011); Portugal	Hydration	79·7	14·6	n/a		n/a		n/a		Neutral
Chia *et al*.^(19)^, (2015); Singapore	Hydration:	44·1	14·0	Pre-exercise: 47·2	22·0	n/a		n/a		Neutral
	Pre-exercise hydration^(9)^, during exercise hydration^(13)^, post-exercise hydration^(8)^			During exercise: 48·7	16·9					
				Post-exercise: 33·2	17·9					
Daniel *et al.* ^(29)^, (2016); Brazil	Macro nutrients, micronutrients, food groups, sport nutrition belief	57·0	9·9	Macro nutrients: 72·0, micronutrients: 27·0, food groups: 98·9, sport nutrition beliefs 70·9	16·6	n/a		n/a		Neutral
					22·6					
					3·5*					
					15·9					
Escribano-Ott *et al*.^(30)^ (2021); Spain	Nutrients, hydration, recovery, weight management, supplements	42·8^†^		Nutrients: 51·5, hydration: 47·2, recovery: 41·9, mass management: 47·7, supplementation: 25·7	16·1	Non-pro: 49·9^†^		Non-pro: Nutrients: 59·5, hydration: 57·9, recovery: 55·2, mass management: 51·4, supplementation: 25·3 Pro: Nutrients: 51·2, hydration: 51·8, recovery: 45·0, mass management: 47·9, supplementation: 33·8	11·7	Neutral
					18·8				13·9	
					1·71	Pro: 46·0^†^			14·9	
					10·06				7·1	
					22·5				19·9	
									18·4	
									24·7	
									20·4	
									17·3	
									27·3	
Foo *et al*.^(31)^, (2021); UK	Energy and refuelling, hydration, supplements, protein	68·3		Energy and refuelling: 81·0, hydration: 68·9, supplements: 66·7, protein: 40·0		n/a		n/a		Neutral
Gonçalves *et al*.^(32)^, (2014); Brazil	GNK, food pyramid	59·9	18	NR		n/a		n/a		Neutral
Hardy *et al.* ^(33)^, (2017); USA	Dietary recommendations, nutrients, food choice, diet–disease relationships	58·1	0·9^†^	^†^Dietary recommendations: 62·7, nutrients: 60·0, food choice: 57·1, diet–disease relationships: 47·1	0·9	58·7	0·9^†^	^†^Dietary recommendation: 65·5, nutrients: 60·2, food choice: 61·4, diet–disease relationships: 47·1	0·9	Neutral
					0·9				1·0	
					1·9				1·7	
					1·6				1·9	
Heikkilä *et al*.^(35)^, (2018); Finland	Nutrition recommendations, supplements, hydration, recovery, food choice/body image	72·7	8·8	Nutrition recommendations: 69·2 , supplements: 66·0, hydration: 85·2, recovery: 72·8, food choice/body image: 84·0	10·4	80·8	8·7	Nutrition recommendations: 76·8, supplements: 79·8, hydration: 90·9, recovery: 82·0, food choice/body image: 91·4	10·8	Neutral
					22·6				20·0	
					15·3				10·9*	
					13·0				11·8	
					14·7				9·9*	
Heikkilä *et al.* ^(34)^, (2019); Finland	Nutrition recommendations, supplements, hydration, recovery, food choice/body image	Total participants: 77·7	7·6	*Group 1:* Nutrition recommendations: 75·8, supplements: 71·4, hydration: 87·2, recovery: 77·3, food choice/body image: 83 *Group 2:* Nutrition recommendations: 75·2, supplements: 73·5, hydration: 87·8, recovery: 77·9, food choice/body image: 87·9		n/a		n/a		Positive
Jusoh^(36)^, (2014); Malaysia	Hydration	F:73·6	6·311·7	NR		n/a		n/a		Neutral
		M:71·8								
Jusoh *et al*.^(37)^, (2021); Malaysia	GNK, SNK	F: 78·6	13·1	n/a		n/a		n/a		Positive
		M: 77·3	12·1							
Kettunen *et al.* ^(38)^ (2021); Finland	Nutrition recommendations, supplements, hydration, recovery, food choice/body image	76·0	7·3	Nutrition recommendations: 73·4, supplements: 67·4, hydration: 92·5, recovery: 71·8, food choice/body image: 87·1	8·4	n/a		n/a		Neutral
					19·1					
					10·0					
					14·6					
					11·3					
Laramée *et al*.^(39)^ (2017); Canada	Carbohydrates^(8)^, proteins^(9)^, lipids^(8)^, SNK^(7)^	Group 1:60·8		NR		n/a		n/a		Neutral
		Group 2:51·4								
Mandic *et al*.^(40)^, (2013); Croatia/Serbia	SNK	30·9	16·1	NR		45·2	17·3	n/a		Neutral
Manore *et al*.^(41)^, (2017); USA	Hydration, protein/carbohydrate, supplements, food choice	45·6		NR		n/a		n/a		Neutral
Nascimento *et al.* ^(42)^, (2016); Brazil	GNK^(3)^, BFP^(1)^, SNK^(10)^	73·6		GNK: 92, BFP: 37, SNK: 83·3		70·0		GNK: 89·2, BFP: 28·4, SNK: 84·5		Neutral
Noronha *et al*.^(43)^, (2020); Brazil	GNK^(3)^, BFP^(1)^, SNK^(10)^	54·6	13·6	GNK: 75·7	22·6	n/a		n/a		Neutral
				BFP: 16·3	9·2					
				SNK: 67·8	21·1					
Philippou *et al*.^(9)^, (2017); Cyprus	Macronutrients/micronutrients^(9)^, Mediterranean diet^(1)^	70·0		NR		n/a		n/a		Neutral
Sanchez-Diaz *et al.* ^(44)^, (2021); Spain	GNK	42·0		NR		n/a		n/a		Neutral
Saribay and Kirbay^(45)^, (2019); Turkey	Dietary recommendations^(9)^, food items^(21)^, diet–disease relationship^(8)^	F: 53·8		NR		n/a		n/a		Neutral
		M: 52·6								
Spendlove *et al*.^(46)^, (2012); Australia	Dietary recommendations, nutrients, food choice, diet–disease relationship	57·6		Dietary recommendations; 65·4, nutrients: 60·9, food choice: 60·0, diet-disease relationship 45·0		CM: 63·1		CM: Dietary recommendations 65·4, nutrients 67·7, food choice 64·0, diet–disease relationship 50·0		Neutral
						DT: 86·2		DT: Dietary recommendations77·7, nutrients 92·0, food choice 76·0, diet–disease relationship 83·5		
Supriya & Ramaswami^(47)^, (2013); India	GNK, performance, food groups, carbohydrates, proteins, antioxidants, sports drinks, hydration	52·6	15	GNK 92, performance: 39 food groups: 42, carbohydrate: 22, proteins: 81, antioxidants: 58, sports drink: 26, hydration: 42		n/a		n/a		Neutral
Walsh *et al.* ^(57)^, (2011); Ireland	Hydration, supplements, energy/refuelling, protein	59·6	12·8	Hydration: 76·4, supplements 69·3, energy/refuelling 57·1, protein 39·2	20·7	n/a		n/a		Neutral
					28·6					
					15·4					
					31·4					
Webb and Beckford^(49)^, (2014); Trinidad and Tobago	GNK	52·2	13·8^†^	NR		n/a		n/a		Neutral
Wyon *et al*.^(20)^ (2014); UK	GNK	F: 37·1^‡^		NR		F:51·0		NR		Neutral
		M:37·0^‡^				M:54·0				

M, male; F, female; *n*, number; GNKQ, general nutrition knowledge questionnaire; GNK, general nutrition knowledge; SNK, sports nutrition knowledge; BFP, Brazilian food pyramid; NR, not reported; GeSNK, general and sports nutrition knowledge; DRI, dietary reference intake.

* sd reported as crossing 100 %, reported as stated by original author.

† Review author calculated mean total % based on mean score reported.

‡ Author calculated pooled results due to stratification by multiple factors.

A total of 4553 adolescent athletes were included, comprised of 1871 females, 2500 males and 182 either not reported or unable to be separated from the comparison group data. The mean age ranged from 12·7 to 18·9 years. The sample sizes ranged from 10 to 586 participants. A non-adolescent athlete comparison group of adult athletes^(20,23,30,33,42)^, non-athletic adults^(46)^, coaches^(35,40)^ or non-athlete adolescents^(27)^ was used in nine studies and included 574 participants, comprising 205 males, 242 females and 127 whose gender was not reported. The comparison group age ranged from 16·7 to 44·3 years old. Three studies did not report the specific sports played by the study population. Eighteen studies recruited participants from one type of sport including swimming^(9,22,31,49)^, basketball^(28,30,44)^, soccer^(41,43)^, gymnastics^(21)^, ballet^(20)^, rugby^(57)^, table tennis^(23)^, American football^(24)^, cross-country skiing^(38)^, handball^(37)^, volleyball^(29)^ and synchronised swimming^(40)^. The remaining studies represented various sports, including track and field^(47)^, aesthetic sports^(39)^, endurance sports^(34,35)^ and various team and individual sports^(25–27,36,42,45,46)^. Sporting levels included competitive^(9,21–23,26,30,32,34,35,39,40,42,49)^, regional/state/national competitors^(27–29,36–38,43,47)^, high school/college^(19,24,27,33,41,45,57)^, elite^(20,44,46)^ and one study did not report sporting level^(25)^. NK was assessed using twenty-one questionnaires across thirty-two studies. Twenty articles assessed a combination of two or more of the following: GNK, SNK, hydration and supplements. The remaining articles (*n* 12) assessed only one aspect (GNK (*n* 8), SNK (*n* 1) and hydration (*n* 3)). The number of questions ranged from 10 to 113; half of the studies (*n* 16) used a questionnaire with twenty five or fewer questions, ten studies used more than twenty-five questions and four did not report the total number assessed.

### Risk of bias within studies

Detailed quality assessments for all included studies, with scores assigned for each validity criterion of the Academy of Nutrition and Dietetics ‘Quality Criteria Checklist: Primary Research’^(52)^, are available in the supplementary material (online Supplementary Table S4). Only two studies received an overall ‘positive’ rating^(34,37)^, with all remaining studies (*n* 30) receiving ‘neutral’ ratings, indicating a moderate quality level. The main reasons for neutral ratings were related to the selection of study subjects (criterion 2) and the use of valid and reliable measurement tools (criterion 7) followed by an inadequate description of study settings (criterion 6).

Most studies (*n* 28) described the demographic characteristics required by this review (age, gender and sport played); however, few (*n* 4) studies addressed whether the sample population was representative of the broader target population. Four studies reported the use of convenience sampling, which may not have provided a representative study population. The remaining studies often had small sample sizes, with nineteen studies containing fewer than 100 participants. Of the studies with more than 100 participants (*n* 13), seven were recruited from a variety of sports without clarifying which population the sample should have represented. Ten studies were recruited from just one sports club, sports academy, high school or sports team.

Validation of the questionnaire used was reported in twenty-nine studies; most (*n* 13) described the tool as a ‘previously validated questionnaire’, and few (*n* 2) revalidated it after modifying the questionnaire used. Two studies^(31,41)^ reported using a previously validated questionnaire, but when cross-checked with the cited article^(57)^ from which the questionnaire was used, it was found that the tool had not been validated, and the lack of reliability and validity testing was declared as a limitation of the study.

Most studies (*n* 20) provided an adequate description of the study settings and protocols. However, seven studies failed to adequately describe the method of administering the questionnaire; for example, where the questionnaire was administered, whether it was handwritten or electronic, self-administered or interviewer administered. Five studies did not provide general details such as where the assessments took place.

### Results of individual studies

#### Subsection comparison

##### General nutrition knowledge and sports nutrition knowledge

Twenty-six studies assessed GNK, including subsections on dietary recommendations, macro- and micronutrients, food choices, diet–disease relationships and food groups/food pyramid. Sixteen studies assessed SNK; most studies (*n* 15) assessed SNK in conjunction with other NK domains and only one assessed SNK as a standalone subject. The subtopics included under SNK were performance, recovery, energy/refuelling, supplements and hydration. Four studies compared GNK to SNK, and in all cases, GNK was the highest scoring of the two sections.

Supplement knowledge was assessed as a subtopic in eight of the included studies, and no study assessed supplements as a standalone topic. Three different questionnaires were used in eight studies. Three studies^(34,35,38)^ using the Heikkila *et al*.^(58)^ questionnaire reported supplements to be the lowest scoring section. Likewise, Escribano-Ott *et al*.^(30)^ reported supplements as the lowest-scoring subsection, and Bird and Rushton^(26)^ described the supplement section as the greatest source of uncertainty, with more ‘don’t know’ responses than any other section (45 %). Similarly, two studies^(26,30)^ that used questionnaires based on Zinn *et al.* ^(59)^ reported poor scores.

##### Hydration

Hydration knowledge was assessed in fourteen studies. Eleven studies assessed hydration knowledge in combination with other NK domains, with three studies exclusively assessing hydration knowledge^(19,28,36)^. Chia *et al.* ^(19)^ created sub-sections within hydration and found that post-exercise hydration received the lowest scores (mean % correct = 33·2 %), while pre- and post-exercise hydration mean % correct scores were similar at 47·2 % and 47·7 %, respectively.

Of the studies assessing hydration in conjunction with other NK domains, only nine reported subsection scores. Four of these studies reported hydration as the highest-scoring subsection (hydration mean score = 85·8 %), three of which utilised the same questionnaire and assessed a similar population of endurance athletes in Finland (hydration mean score = 88·2 %)^(34,35,38)^. Altavilla *et al*.^(22)^ reported a mean hydration score lower than the total mean score on the NK questionnaire. The remaining four studies reported hydration knowledge as average when compared with other topics.

#### Comparison across questionnaires (between studies)

##### General nutrition knowledge questionnaire

The General nutrition knowledge questionnaire was validated in an adult (non-athlete) population in the UK and contains 110 items related to dietary recommendations, sources of nutrients, choosing everyday foods and diet–disease relationships^(60)^. Four studies included in this review used this questionnaire^(20,21,33,46)^. Aguilo *et al*.^(21)^ made major modifications to the questionnaire, resulting in only sixty-one questions being included. The other three studies did not report modifications or made minor modifications.

The mean % correct scores ranged from 37·0 % to 58·1 %^(20,33)^. Subsection scores were reported by two studies^(33,46)^ both reported the lowest scoring section to be diet-disease relationships and the highest scores in dietary recommendations for adolescent athlete groups.

##### Leite et al., (2016)

Leite *et al.* ^(61)^ validated a NK questionnaire based on previous studies^(32,62)^. The questionnaire contains fourteen questions in three sections: GNK, SNK, and the Brazilian Food Pyramid. Three Brazilian studies used this tool to assess NK in adolescent athletes, none of which reported modifying the previously validated questionnaire.

The mean score of adolescent athletes ranged from 54·6 %^(43)^ to 73·6 %^(42)^. The highest scores in all three studies for both adolescents and adults were related to GNK, and the lowest were found in Brazilian Food Pyramid. Noronha *et al*.^(43)^ did not compare the results to a non-adolescent group, while Argolo *et al*.^(23)^ and Nascimento *et al*.^(42)^ compared adolescent athletes with adult athletes who participated in the same categories of sports. Adult athletes’ mean score correct was reported as 66·7 %^(23)^ and 70 %^(42)^.

##### Walsh et al., (2011)

Walsh *et al*.^(57)^ created a GNK questionnaire to assess NK in adolescent athletes in Ireland. The questionnaire contains sixteen questions that were compiled from both validated and unvalidated questionnaires regarding hydration, supplements, energy/refuelling and proteins. The questionnaire was pilot tested for comprehension but did not undergo further psychometric testing to determine its validity and reliability. Three studies used this questionnaire, including the author of the questionnaire^(31,41,57)^. Manore *et al*.^(41)^ reported that the questionnaire contained twelve questions that may have been an unreported deviation from the original questionnaire or related to counting sub-questions differently than the original author.

The results showed a range of mean % correct scores from 45·6 %^(41)^ to 68·3 %^(31)^. Subsection scores were reported in two studies^(31,57)^, which both indicated the lowest scoring section to be ‘protein’ and the highest ‘energy/refuelling’.

##### Heikkila et al. (2018)

Heikkilä *et al*.^(58)^ developed a seventy-nine-item questionnaire assessing NK topics: nutrition recommendations, supplements, hydration, recovery and the association between food choice and body image. The questionnaire has been validated in a population of endurance athletes in Finland. All three articles that used this tool also assessed NK in Finnish athletes in various endurance sports^(34,35,38)^. Heikkilä *et al*.^(34)^ removed one question considered inappropriate for the target population, with no modifications reported in the other two studies.

The results across adolescent athletes were comparable, with total mean correct reported as 72·7 %^(34)^, 76 %^(38)^ and 77·7 %^(35)^. One study included an adult comparison group and reported a mean total correct of 80·8 %^(35)^.

##### Zinn et al. (2005)

Zinn *et al*.^(59)^ created and validated an eighty-four-item SNK questionnaire covering the following subtopics: nutrients, dietary reference intake, recovery, weight gain, weight loss and supplements. Two studies utilised this questionnaire and did not report modifying it for use; however, the number of questions reported was inconsistent with the original questionnaire with Bird and Rushton^(26)^ using a ninety-question version and Escribano-Ott *et al*.^(30)^ using a twenty-three question version, which used the subsections from the original questionnaire but reported a reduced number for each subsection. Despite the inconsistency in the number of questions reported, the total mean percentage of correct answers was comparable at 42·8 %^(30)^ and 43·8 %^(26)^.

##### Nichols et al. (2005)

Nichols *et al*.^(63)^ created a seventeen-item questionnaire that was pilot-tested on college soccer players. Two studies reported the use of this questionnaire with modifications. Jusoh^(36)^ reduced the total number of questions to 10 and pilot-tested the new version in schoolchildren. Carvalho *et al*.^(28)^ used a sixteen-item Portuguese version of the questionnaire and reported lower internal consistency after modification.

Despite the modifications to the questionnaire, the results were somewhat comparable with Jusoh^(36)^ reported mean correct scores for females and males at 73·6 % and 71·8 %, respectively, and Carvalho *et al*.^(28)^ who reported mean correct scores for the total group at 79·7 %.

#### Nutrition knowledge scores of adolescent athletes *v*. comparison group (within studies)

Nine studies included a non-adolescent athlete comparison group, including adult athletes (*n* 4), coaches (*n* 2), adult non-athletes (*n* 1) and adolescent non-athletes (*n* 1).

Two studies^(23,42)^ comparing adolescent athletes with adult athletes within the same sports category used the same questionnaire^(61)^. Argolo *et al*.^(23)^ reported statistically significant higher overall scores in the adult comparison group (66·7 % *v*. 55 %, *P* < 0·5) than in the adolescent athlete group; however, the adult athletes scored lower within the GNK subsection than the adolescents. Conversely, Nascimento *et al*.^(42)^ reported lower overall scores in adult athletes (70·0 %) than in adolescent athletes (73·6 %). Hardy *et al*.^(33)^ compared 18–19-year-old athletes to athletes aged ≥ 20 years with similar total and subsection results (total mean correct 58·1 % and 58·7 %, respectively). The remaining four studies using adult comparison groups (adult athletes, adult non-athletes and coaches) reported higher total mean correct scores in adult comparison groups.

Calella *et al*.^(27)^ compared athletes representing team sports (volleyball), aesthetic sports (gymnastics) and endurance sports (swimming) with adolescent gym members and inactive adolescents. All athlete groups scored higher on mean total knowledge scores in all subsections than non-athletes.

## Discussion

This review summarised papers on NK in adolescent athletes published since 2010. The key findings were as follows: (1) NK mean scores ranged from 33·3 % to 90·6 %, covering a broad range of topics related to GNK and SNK; however, the heterogeneity of the NK assessment tools used creates difficulty in making definitive statements on NK levels. (2) The areas of strength and weakness within NK topics were not consistent among studies, except where studies directly compared GNK and SNK, and it was reported that GNK scores were higher. And for supplement score, where all studies reporting a supplement sub-section reporting that scores were poor for this topic. (3) The quality rating of all but two studies received a ‘neutral’ rating with the main areas of weakness being an inadequate description of study participant selection and lack of clarity regarding the validity and reliability of data collection tools used. (4) Athletes assessed were most commonly from European countries, and multiple sporting types were often included within an individual study, of which swimming and basketball were the most reported athletes.

The NK assessment tools used varied between studies, making it difficult to directly compare the results. When the same NK assessment tool was used across multiple studies, direct comparison of results was not always possible as the individual studies often modified the questionnaire from the original form to meet the aims of the specific study, as seen in previous reviews^(14,17)^. While all NK assessment tools provided a quantitative NK score, the topics covered varied between studies. Some tools were designed to assess only a single area of NK, such as GNK^(20,44,49)^ or hydration^(28,36)^ making comparisons between these studies illogical because of the differences in NK domains assessed. Furthermore, the level of difficulty of NK’s assessment tools has rarely been ascertained, as most studies did not provide the questionnaire used. Previous literature reviews^(14,17,18)^ assessing the NK of athletes have similarly reported difficulty in comparing results between studies due to the heterogeneity of NK assessment tools used. It has been suggested that a greater level of insight may be obtained within individual studies using a comparison group to benchmark the results^(18)^. Despite these recommendations to benchmark results, in the current review, only eight studies compared adolescent athletes with adult athletes^(20,23,30,33,42)^, coaches^(35,40)^ and non-athlete adults^(46)^ with only one study using an age-matched comparison group^(27)^. Adult athletes scored numerically higher than adolescent athletes in all but one study^(32)^ that compared these groups, which is expected based on previous reports that age is associated with higher NK, likely because older individuals have higher levels of education and life experience^(23)^. When an age-matched non-athlete group was used^(27)^, adolescent athletes scored higher in all sections (total NK, GNK and SNK), indicating that there may be a need for future research to include adolescent non-athlete comparison groups to gauge the level of understanding between athletes and non-athletes.

As per previous related reviews, the heterogeneity of the tools used and the subdomains of NK assessed create difficulty in identifying specific areas of strength or weakness. However, adolescent athletes consistently scored higher in the GNK than in the SNK, where the two were directly compared within studies^(23,27,42,43)^. This finding differs from previous literature reviews, which found mixed results when measuring GNK *v*. SNK in adult athletes^(14)^. Studies that assessed and reported supplements as a subtopic indicated poor understanding, this is concerning as the use of supplements without recommendations by professionals such as accredited practicing sports dietitians or medical doctors is widespread, despite the risks of contamination with banned substances and unintended health side effects^(1,64,65)^.

Most studies were considered to have a ‘neutral’ quality rating because of a lack of clarity regarding the study subject selection and the validation of the NK assessment tool used. Nineteen studies reported fewer than 100 participants and rarely justified the study sample size. Furthermore, there was a lack of detail regarding the target population of the study was intending to represent. Previous reviews on adult athletes also reported limitations within studies related to inadequate statistical reporting and failure to use validated NK questionnaires^(17)^, with small sample sizes also common in studies on adult athletes. Of note, research among adolescents comes with obstacles that may make recruitment difficult: first, identifying a group that is large enough to provide meaningful results. This is followed by a complex process of recruiting adolescents and obtaining consent from their parent/guardian. Additionally, it is essential that researchers are sensitive to the target population and potential vulnerability; for example, the pressure to complete an NK assessment may be a concern for those at risk of disordered eating patterns due to the topic of the assessment and the known triggers of stress and negative emotions in body image dissatisfaction and disordered eating^(66,67)^. Regardless of the barriers faced in recruitment, researchers must describe the method of recruitment and provide some insight into the broader community they are attempting to represent, so that the results may be interpreted accurately. A further concern regarding the quality of the articles was the lack of clarity regarding the validity of the NK assessment tools used. Several studies reported using validated questionnaires; however, upon further examination, it was determined that the questionnaire used was modified from a previously validated questionnaire without revalidation attempts, insufficiently validated or incorrectly declared as validated. The use of a tool that has not been validated in the target population may bring into question the accuracy of the results presented.

The athletes assessed were mostly from European countries, differing from previous reviews of adult athletes^(14,17)^ which found most studies from North America primarily from the USA. This may be because American collegiate athletes are convenient to recruit, and thus represent a large proportion of studies on adult athletes in Western countries. Conversely, adolescent athletes are not typically grouped at the scale of collegiate athletes, and the need for parental consent to participate in research studies may prove an obstacle to recruiting large numbers. In this review, several regions were underrepresented, with Brazil being the only country included from South America, Australia the only country from Oceania and no countries included from Africa. The lack of representation in African countries appears disproportionate when considering the number of athletes from this region; for example, endurance running is dominated by Ethiopian and Kenyan athletes^(68)^. The lack of funding in this region may explain the absence of studies in this region. Thirty-five different sports were reported throughout the studies; however, only one study involved many sports. Furthermore, nine studies recruited participants from at least six different sports, resulting in the total number of participants in these sports being limited, and their representation unclear. Three studies did not report the type of sports played, which brings challenges in interpreting the generalisability of the results to other adolescent athlete groups.

### Recommendations and future research

#### Implications for practice and policy

The results of this literature review indicate that there is a need for heightened education regarding sports nutrition for adolescent athletes. Considering the impact on both long-term health and athletic performance, there is great benefit to be reaped from the implementation of nutrition education programmes for athletes of all levels. A systematic literature review^(13)^ reported the effectiveness of nutrition education interventions for athletes, however, was unable to recommended method of providing nutrition education to athletes due to poor validation of interventions and inconsistent delivery methods. However, sports nutrition education interventions for athletes may be improved by using methods such as co-design and implementing technology and social media^(69)^.

#### Implications for future research

The findings from this review suggest that there is a need to improve the tools used to assess NK in adolescent athletes. Innumerable NK assessment tools are currently used worldwide, and future research would benefit from the validation of a comprehensive NK assessment tool in a range of sporting types and culturally diverse settings. The creation and use of such a tool would provide great benefit to the research community and aid the direct comparison of results between studies. Additionally, there is a lack of studies from African countries, this an area of possible future research due to the high number of sports played throughout the African continent.

### Limitations

#### Limitations of studies

The greatest limitations of the studies were small sample sizes, lack of justification for sample size or description of the target population and the use of unvalidated or insufficiently validated tools. Several authors were contacted to clarify inconsistencies or provide missing data; however, none of the authors responded to the request for information. As such, it is possible that some outcomes or characteristics were not fully represented in this review. Few studies included comparison groups, particularly age-matched groups; therefore, it is not possible to conclude whether athletes demonstrate higher NK than groups of non-athlete adolescents. Studies rarely reported education level of the adolescents; however, this may be due to the young age of the participants restricting the education level to high school years only.

#### Limitations of review

The main limitation of this review is the implausibility of the meta-analysis owing to the heterogeneity of the NK questionnaires used throughout the studies. There was a risk of publication bias due to the exclusion of studies published before 2010, which included eleven studies that would otherwise have met the inclusion criteria. However, considering that nutrition education and assessment of NK is an evolving area of interest, it is proposed that the articles included in the current review are more likely to provide insight into the current levels of NK based on the modern understanding of nutrition requirements for adolescent athletes. Additionally, of the eleven excluded articles published before 2010 eight were included in a similar previous review^(18)^. Further potential sources of bias arose in the exclusion of non-English language studies and grey literature, and the inability to source four studies for full-text screening. The review also was unable to compare the results of studies based on participant characteristics such as gender or level of sporting ability due to the heterogeneity of the studies and a lack of reporting on differences between groups based on demographic characteristics.

### Conclusion

In conclusion, this review found that adolescent athletes showed strength in GNK when compared with SNK. Adolescent athletes exhibited uncertainty regarding the use and regulation of supplements. However, other areas of strength and weakness in NK were difficult to identify owing to the heterogeneous nature of the NK assessment tools used. There is a need to consistently utilise NK tools that are validated appropriately for the target population to strengthen the results. Furthermore, the increased use of comparison groups, particularly age-matched groups, may enhance the ability to interpret results with confidence.

## Supporting information

Hulland et al. supplementary material 1 Hulland et al. supplementary material

Hulland et al. supplementary material 2 Hulland et al. supplementary material

Hulland et al. supplementary material 3 Hulland et al. supplementary material

Hulland et al. supplementary material 4 Hulland et al. supplementary material

Hulland et al. supplementary material 5 Hulland et al. supplementary material

## Supplementary material

For supplementary material/s referred to in this article, please visit https://doi.org/10.1017/S0007114523002799

## References

[1] Desbrow B , McCormack J , Burke LM , et al. (2014) Sports dietitians Australia position statement: sports nutrition for the adolescent athlete. Int J Sport Nutr Exerc Metab 24, 570–584. 24668620 10.1123/ijsnem.2014-0031

[2] Fritzen AM , Lundsgaard AM & Kiens B (2019) Dietary fuels in athletic performance. Ann Rev Nutr 39, 45–73. 31136266 10.1146/annurev-nutr-082018-124337

[3] Norris SA , Frongillo EA , Black MM , et al. (2022) Nutrition in adolescent growth and development. Lancet 399, 172–184. 34856190 10.1016/S0140-6736(21)01590-7

[4] Weaver SP , Kelley L , Griggs J , et al. (2014) Fit and healthy family camp for engaging families in a child obesity intervention: a community health center pilot project. Fam Community Health 37, 31–44. 24297006 10.1097/FCH.0000000000000013

[5] Christoph MJ , Larson NI , Winkler MR , et al. (2019) Longitudinal trajectories and prevalence of meeting dietary guidelines during the transition from adolescence to young adulthood. Am J Clin Nutr 109, 656–664. 30831584 10.1093/ajcn/nqy333 PMC6408200

[6] Thomas DT , Erdman KA & Burke LM (2016) Position of the academy of nutrition and dietetics, dietitians of Canada, and the American college of sports medicine: nutrition and athletic performance. J Acad Nutr Diet 116, 501–528. 26920240 10.1016/j.jand.2015.12.006

[7] Burrows T , Harries SK , Williams RL , et al. (2016) The diet quality of competitive adolescent male rugby union players with energy balance estimated using different physical activity coefficients. Nutrients 8, 548. 27618089 10.3390/nu8090548 PMC5037533

[8] Martínez-Rodríguez A , Martínez-Olcina M , Hernández-García M , et al. (2021) Mediterranean diet adherence, body composition and performance in beach handball players: a cross sectional study. Int J Environ Res Public Health 18, 2837. 33802192 10.3390/ijerph18062837 PMC7999029

[9] Philippou E , Middleton N , Pistos C , et al. (2017) The impact of nutrition education on nutrition knowledge and adherence to the Mediterranean diet in adolescent competitive swimmers. J Sci Med Sport 20, 328–332. 27692575 10.1016/j.jsams.2016.08.023

[10] Zanella PB , August PM , Alves FD , et al. (2019) Association of healthy eating index and oxidative stress in adolescent volleyball athletes and non-athletes. Nutrition 60, 230–234. 30682544 10.1016/j.nut.2018.10.017

[11] Birkenhead KL & Slater G (2015) A review of factors influencing athletes’ food choices. Sports Med 45, 1511–1522. 26243016 10.1007/s40279-015-0372-1

[12] Trakman GL (2018) The development and validation of the nutrition for sport knowledge questionnaire (NSKQ) and abridged nutrition for sport knowledge questionnaire (A-NSKQ) to investigate the sports nutrition knowledge of Australian athletes. Doctoral Thesis, La Trobe University Research Online, La Trobe University.

[13] Tam R , Beck KL , Manore MM , et al. (2019) Effectiveness of education interventions designed to improve nutrition knowledge in athletes: a systematic review. Sports Med 49, 1769–1786. 31372860 10.1007/s40279-019-01157-y

[14] Trakman G , Forsyth A , Devlin B , et al. (2016) A systematic review of athletes’ and coaches’ nutrition knowledge and reflections on the quality of current nutrition knowledge measures. Nutrients 8, 570. 27649242 10.3390/nu8090570 PMC5037555

[15] Devlin BL & Belski R (2015) Exploring general and sports nutrition and food knowledge in elite male Australian athletes. Int J Sport Nutr Exerc Metab 25, 225–232. 25387042 10.1123/ijsnem.2013-0259

[16] Thakur S & Mathur P (2021) Nutrition knowledge and its relation with dietary behaviour in children and adolescents: a systematic review. Int J Adolesc Med Health 34, 381–392. 33594848 10.1515/ijamh-2020-0192

[17] Janiczak A , Devlin BL , Forsyth A , et al. (2022) A systematic review update of athletes’ nutrition knowledge and association with dietary intake. Br J Nutr 128, 1156–1169. 34706784 10.1017/S0007114521004311

[18] Heaney S , O’Connor H , Michael S , et al. (2011) Nutrition knowledge in athletes: a systematic review. Int J Sport Nutr Exerc Metab 21, 248–261. 21719906 10.1123/ijsnem.21.3.248

[19] Chia M , Mukherjee S & Huang D (2015) Thirst for drink knowledge: how Singaporean youth athletes measure up in an exercise hydration knowledge questionnaire. Int J Sports Sci Coach 10, 841–850.

[20] Wyon MA , Hutchings KM , Wells A , et al. (2014) Body mass index, nutritional knowledge, and eating behaviors in elite student and professional ballet dancers. Clin J Sport Med 24, 390–396. 24326932 10.1097/JSM.0000000000000054

[21] Aguilo A , Lozano L , Tauler P , et al. (2021) Nutritional status and implementation of a nutritional education program in young female artistic gymnasts. Nutrients 13, 1399. 33919356 10.3390/nu13051399 PMC8143314

[22] Altavilla C , Prats-Moya MS & Perez PC (2017) Hydration and nutrition knowledge in adolescent swimmers. Does water intake affect urine hydration markers after swimming? Int J Appl Exerc Physiol 6, 37–45.

[23] Argôlo D , Borges J , Cavalcante A , et al. (2018) Poor dietary intake and low nutritional knowledge in adolescent and adult competitive athletes: a warning to table tennis players. Nutr Hosp 35, 1124–1130. 30307296 10.20960/nh.1793

[24] Atkins WC , McDermott BP , Koji K , et al. (2021) Effects of hydration educational intervention in high school football players. J Strength Cond Res 35, 385–390. 33337701 10.1519/JSC.0000000000003866

[25] Bakhtiar M , Masud-ur-Rahman M , Kamruzzaman M , et al. (2021) Determinants of nutrition knowledge, attitude and practices of adolescent sports trainee: a cross-sectional study in Bangladesh. Heliyon 7, e06637. 33898807 10.1016/j.heliyon.2021.e06637 PMC8056407

[26] Bird SP & Rushton BD (2020) Nutritional knowledge of youth academy athletes. BMC Nutr 6, 35. 32821418 10.1186/s40795-020-00360-9 PMC7433089

[27] Calella P , Gallè F , Di Onofrio V , et al. (2021) Gym members show lower nutrition knowledge than youth engaged in competitive sports. J Am Coll Nutr 40, 465–471. 32758109 10.1080/07315724.2020.1792375

[28] Carvalho P , Oliveira B , Barros R , et al. (2011) Impact of fluid restriction and ad libitum water intake or an 8 % carbohydrate-electrolyte beverage on skill performance of elite adolescent basketball players. Int J Sport Nutr Exerc Metab 21, 214–221. 21719902 10.1123/ijsnem.21.3.214

[29] Daniel NVS , Jurgensen LP , Padovani RD , et al. (2016) Impact of an interdisciplinary food, nutrition and health education program for adolescent Brazilian volleyball players. Rev Nutr 29, 567–577.

[30] Escribano-Ott I , Mielgo-Ayuso J & Calleja-González J (2021) A glimpse of the sports nutrition awareness in Spanish basketball players. Nutrients 14, 27. 35010902 10.3390/nu14010027 PMC8746623

[31] Foo WL , Faghy MA , Sparks A , et al. (2021) The effects of a nutrition education intervention on sports nutrition knowledge during a competitive season in highly trained adolescent swimmers. Nutrients 13, 2713. 34444873 10.3390/nu13082713 PMC8400374

[32] Gonçalves CB , Nogueira JAD & Da Costa THM (2014) The food pyramid adapted to physically active adolescents as a nutrition education tool. Rev Bras Cienc Esporte 36, 29–44.

[33] Hardy R , Kliemann N , Evansen T , et al. (2017) Relationship between energy drink consumption and nutrition knowledge in student-athletes. J Nutr Educ Behav 49, 19–26. 27720600 10.1016/j.jneb.2016.08.008

[34] Heikkilä M , Lehtovirta M , Autio O , et al. (2019) The impact of nutrition education intervention with and without a mobile phone application on nutrition knowledge among young endurance athletes. Nutrients 11, 2249. 31540535 10.3390/nu11092249 PMC6770376

[35] Heikkilä M , Valve R , Lehtovirta M , et al. (2018) Nutrition knowledge among young Finnish endurance athletes and their coaches. Int J Sport Nutr Exerc Metab 28, 522–527. 29252046 10.1123/ijsnem.2017-0264

[36] Jusoh N (2014) Relationship between hydration status, hydration knowledge and fluid intake behaviour among school athletes of selected Perak sport schools. J Sports Sci Phys Educ 3, 11–19.

[37] Jusoh N , Lee JLF , Tengah RY , et al. (2021) Association between nutrition knowledge and nutrition practice among Malaysian adolescent handball athletes. Mal J Nutr 27, 279–291.

[38] Kettunen O , Heikkilä M , Linnamo V , et al. (2021) Nutrition knowledge is associated with energy availability and carbohydrate intake in young female cross-country skiers. Nutrients 13, 1769. 34067303 10.3390/nu13061769 PMC8224650

[39] Laramée C , Drapeau V , Valois P , et al. (2017) Evaluation of a theory-based intervention aimed at reducing intention to use restrictive dietary behaviors among adolescent female athletes. J Nutr Educ Behav 49, 497–504. 28601167 10.1016/j.jneb.2017.03.009

[40] Mandic GF , Peric M , Krzelj L , et al. (2013) Sports nutrition and doping factors in synchronized swimming: parallel analysis among athletes and coaches. J Sports Sci Med 12, 753–760. 24421736 PMC3873667

[41] Manore MM , Patton-Lopez MM , Meng Y , et al. (2017) Sport nutrition knowledge, behaviors and beliefs of high school soccer players. Nutrients 9, 350. 28368321 10.3390/nu9040350 PMC5409689

[42] Nascimento M , Silva D , Ribeiro S , et al. (2016) Effect of a nutritional intervention in athlete’s body composition, eating behaviour and nutritional knowledge: a comparison between adults and adolescents. Nutrients 8, 535. 27618088 10.3390/nu8090535 PMC5037522

[43] Noronha DC , Santos M , Santos AA , et al. (2020) Nutrition knowledge is correlated with a better dietary intake in adolescent soccer players: a cross-sectional study. J Nutr Metab 2020, 3519781. 31998535 10.1155/2020/3519781 PMC6964714

[44] Sánchez-Díaz S , Yanci J , Raya-González J , et al. (2021) A comparison in physical fitness attributes, physical activity behaviors, nutritional habits, and nutritional knowledge between elite male and female youth basketball players. Front Psychol 12, 685203. 34135836 10.3389/fpsyg.2021.685203 PMC8201790

[45] Saribay AK & Kirbaş Ş (2019) Determination of nutrition knowledge of adolescents engaged in sports. Univ J Educ Res 7, 40–47.

[46] Spendlove JK , Heaney SE , Gifford JA , et al. (2012) Evaluation of general nutrition knowledge in elite Australian athletes. Br J Nutr 107, 1871–1880. 22018024 10.1017/S0007114511005125

[47] Supriya V & Ramaswami L (2013) Knowledge, attitude and dietary practices of track and field athletic men and women aged 18–22. Int J Innov Res Dev 2, 399–404.

[48] Walsh AM , Madigan S , Cleary J , et al. (2013) The nutrition knowledge and weight-making practices of elite and non-elite Irish youth boxers. Proc Nutr Soc 72, E179.

[49] Webb MC & Beckford SE (2014) Nutritional knowledge and attitudes of adolescent swimmers in Trinidad and Tobago. J Nutr Metab 2014, 506434. 24669316 10.1155/2014/506434 PMC3942200

[50] Page MJ , McKenzie JE , Bossuyt PM , et al. (2021) The PRISMA 2020 statement: an updated guideline for reporting systematic reviews. BMJ 372, n71. 33782057 10.1136/bmj.n71 PMC8005924

[51] Singh JA , Siddiqi M , Parameshwar P , et al. (2019) World Health Organization guidance on ethical considerations in planning and reviewing research studies on sexual and reproductive health in adolescents. J Adolesc Health 64, 427–429. 30904091 10.1016/j.jadohealth.2019.01.008 PMC6496912

[52] Academy of Nutrition and Dietetics (2016) Evidence Analysis Manual: Steps in the Academy Evidence Analysis Process. https://www.andeal.org/vault/2440/web/files/2016_April_EA_Manual.pdf (accessed March 2022).

[53] Bilgic P , Tamer F , Yabanci N , et al. (2014) Exploring the use of text messages for increasing nutrition knowledge and improving dietary practices in teen athletes. FASEB J 28.

[54] Carter K , Nash K , Walden A , et al. (2021) Does the source of nutrition information affect nutrition knowledge. Med Sci Sports Exerc 53, 298.

[55] Spronk I , Heaney SE , Prvan T , et al. (2015) Relationship between general nutrition knowledge and dietary quality in elite athletes. Int J Sport Nutr Exerc Metab 25, 243–251. 25252338 10.1123/ijsnem.2014-0034

[56] Patton-Lopez MM , Manore MM , Branscum A , et al. (2018) Changes in sport nutrition knowledge, attitudes/beliefs and behaviors following a 2-year sport nutrition education and life-skills intervention among high school soccer players. Nutrients 10, 1636. 30400200 10.3390/nu10111636 PMC6266993

[57] Walsh M , Cartwright L , Corish C , et al. (2011) The body composition, nutritional knowledge, attitudes, behaviors, and future education needs of senior schoolboy rugby players in Ireland. Int J Sport Nutr Exerc Metab 21, 365–376. 21799215 10.1123/ijsnem.21.5.365

[58] Heikkila M , Valve R , Lehtovirta M , et al. (2018) Development of a nutrition knowledge questionnaire for young endurance athletes and their coaches. Scand J Med Sci Sports 28, 873–880. 28975667 10.1111/sms.12987

[59] Zinn C , Schofield G & Wall C (2005) Development of a psychometrically valid and reliable sports nutrition knowledge questionnaire. J Sci Med Sport 8, 346–351. 16248475 10.1016/s1440-2440(05)80045-3

[60] Parmenter K & Wardle J (1999) Development of a general nutrition knowledge questionnaire for adults. Eur J Clin Nutr 53, 298–308. 10334656 10.1038/sj.ejcn.1600726

[61] Leite MDM , Santos Barbosa Machado AC , Da Silva DG , et al. (2016) Conocimiento sobre alimentacion y nutricion despues del desarrollo de actividades de educacion alimentaria entre ninos y adolescentes deportistas [Knowledge about food and nutrition after the development of food education activities among children and adolescent athletes]. Pensar Prát 19, 56–67.

[62] Zawila LG , Steib CM & Hoogenboom B (2003) The female collegiate cross-country runner: nutritional knowledge and attitudes. J Athl Train 38, 67–74. 12937475 PMC155514

[63] Nichols PE , Jonnalagadda SS , Rosenbloom CA , et al. (2005) Knowledge, attitudes, and behaviors regarding hydration and fluid replacement of collegiate athletes. Int J Sport Nutr Exerc Metab 15, 515. 16327032 10.1123/ijsnem.15.5.515

[64] Mettler S , Lehner G & Morgan G (2022) Widespread supplement intake and use of poor quality information in elite adolescent Swiss athletes. Int J Sport Nutr Exerc Metab 32, 41–48. 34552032 10.1123/ijsnem.2021-0043

[65] Whitehouse G & Lawlis T (2017) Protein supplements and adolescent athletes: a pilot study investigating the risk knowledge, motivations and prevalence of use. Nutr Diet 74, 509–515. 28748643 10.1111/1747-0080.12367

[66] Wells KR , Jeacocke NA , Appaneal R , et al. (2020) The Australian Institute of Sport (AIS) and National Eating Disorders Collaboration (NEDC) position statement on disordered eating in high performance sport. Br J Sports Med 54, 1247–1258. 32661127 10.1136/bjsports-2019-101813 PMC7588409

[67] Naumann E , Svaldi J , Wyschka T , et al. (2018) Stress-induced body dissatisfaction in women with binge eating disorder. J Abnorm Psychol 127, 548–558. 30102065 10.1037/abn0000371

[68] Zani ALS , Gouveia MH , Aquino MM , et al. (2022) Genetic differentiation in East African ethnicities and its relationship with endurance running success. PLoS ONE 17, e0265625. 35588128 10.1371/journal.pone.0265625 PMC9119534

[69] Scharoun L & Mews G (2020) Designed by kids for kids: design strategies for improved outcomes for children’s health and wellbeing in suburban environments. AIP Conf Proc 2230, 040003.

[70] Decher NR , Casa DJ , Yeargin SW , et al. (2008) Hydration status, knowledge, and behavior in youths at summer sports camps. Int J Sports Physiol Perform 3, 262–278. 19211940 10.1123/ijspp.3.3.262

[71] Calella P , Iacullo VM & Valerio G (2017) Validation of a general and sport nutrition knowledge questionnaire in adolescents and young adults: geSNK. Nutrients 9, 439. 28468271 10.3390/nu9050439 PMC5452169

[72] Jürgensen LP , Daniel NVS , Padovani RDC , et al. (2015) Avaliação da qualidade da dieta de atletas de esportes coletivos [Assessment of the diet quality of team sports athletes]. Rev Bras Cineantropometria Desempenho Hum 17, 280.

[73] Lima EDS , Monteiro EADA & Andrade APD (1985) Educação nutricional na escola do primeiro grau em Pernambuco (Brasil): diagnóstico [Nutritional education in primary schools of Pernambuco, Brazil: a diagnosis]. Rev Saúde Publica 19, 508–520. 3939166 10.1590/s0034-89101985000600003

[74] Triches RM & Giugliani ERJ (2005) Obesidade, práticas alimentares e conhecimentos de nutrição em escolares [Obesity, eating practices and nutrition knowledge in schoolchildren]. Rev Saúde Publica 39, 541–547. 16113901 10.1590/s0034-89102005000400004

[75] Sedek R & Yih TY (2014) Dietary habits and nutrition knowledge among athletes and non-athletes in National University of Malaysia (UKM). Pak J Nutr 13, 752–759.

[76] Morissette É , Laramée C , Drapeau V , et al. (2015) Determinants of restrictive dietary behaviors among female high school athletes. Health Behav Policy Rev 2, 378–387.

[77] Kondric M , Sekulic D , Uljevic O , et al. (2013) Sport nutrition and doping in tennis: an analysis of athletes’ attitudes and knowledge. J Sports Sci Med 12, 290–297. 24149808 PMC3761838

[78] Turconi G , Celsa M , Rezzani C , et al. (2003) Reliability of a dietary questionnaire on food habits, eating behaviour and nutritional knowledge of adolescents. Eur J Clin Nutr 57, 753–763. 12792659 10.1038/sj.ejcn.1601607

[79] Öz F , Aydin R , Önsuz M , et al. (2016) Development of a reliable and valid adolescence nutritional knowledge questionnaire. Progr Nutr 18, 125–134.

[80] Ozdoğan Y & Ozcelik AO (2011) Evaluation of the nutrition knowledge of sports department students of universities. J Int Soc Sports Nutr 8, 11. 21892942 10.1186/1550-2783-8-11 PMC3177873

