# Supplementary material for: Adolescent athletes have better general than sports nutrition knowledge and lack awareness of supplement recommendations: a systematic literature review
Source: Br J Nutr. 2023 Dec 6;131(8):1362–76. doi: 10.1017/S0007114523002799 (PMC10950453; doi:10.1017/S0007114523002799)
Supplement: Hulland et al. supplementary material 1 — Hulland et al. supplementary material [file S0007114523002799sup001.docx]

**Article Type:** Systematic Literature Review

**Title:** Adolescent athletes have better general than sports nutrition knowledge and lack awareness of supplement recommendations: A Systematic Literature Review

**Author names:** Susan C. Hulland^1^

Gina L. Trakman^1,2^

Rebekah D. Alcock^1^

**Author Affiliations:**

^1^ Department of Dietetics, Nutrition and Sport, La Trobe University, VIC, Australia.

^2^ Essendon Football Club, VIC, Australia

**Corresponding Author:** Susan Hulland, (23 The Grove, Austinmer, NSW, Australia 2515), +971562635141, [susan@hullandnutrition.com](mailto:susan@hullandnutrition.com)

**Keywords:** Adolescent, athletes, nutrition knowledge

# Introduction

Athletes’ nutrition choices influence sporting performance and recovery post-exercise. Carefully timed pre-, during, and post-training foods and fluids ensure adequate energy availability during exercise and training, aid glycogen re-synthesis post-exercise, and maximise training adaptations ^1, 2^.

The nutritional choices of adolescent athletes are of heightened significance as they face a period of rapid growth and development, during which nutrient intake may influence neurodevelopment, bone mineral density, and the risk of chronic illnesses ^3, 4^. Adhering to population-based dietary guidelines and sport-specific dietary recommendations can provide long-term health benefits and athletic advantages ^1, 5, 6^. Despite the benefits of following dietary recommendations, a study of Australian adolescent rugby players found that players were consuming inadequate vegetable serves, excessive energy was derived from discretionary choices (foods typically high in saturated fat/sodium and/or low in fibre and micronutrients), and carbohydrate intake was insufficient when compared to the Sports Dietitians Australia recommendations^7^. Similarly, low to moderate adherence to recommended dietary guidelines has been observed in adolescent Spanish beach handball players^8^, adolescent Cypriot swimmers^9^, and adolescent Brazilian volleyball players^10^. Considering the implications of non-adherence to both population-based and sport-specific dietary recommendations for short- and long-term health and athletic performance, it is pertinent to investigate the factors influencing adolescent athletes’ food choices.

Birkenhead and Slater’s^11^ theoretical framework describes the influence of multiple factors on athletes’ food choices. Trakman^12^ proposed that these factors may be categorised as modifiable, semi-modifiable, or non-modifiable based on their potential to change when professional guidance and education are provided. No factor can be considered solely responsible for athletes’ food choices; rather, these factors are dynamic, and the level of influence may be situational. Of particular significance is the factor of nutrition knowledge (NK); the level of NK can not only inform nutrition choices, but may also be measured with relative ease compared to using quantitative NK assessment where a numerical score is provided to reflect NK. Furthermore, the introduction of educational intervention programs can result in measurable improvement in this area, as reported in previous systematic literature reviews of athletes’ NK ^13, 14^.

The success of a nutrition education program relies first on an understanding of areas of importance to the targeted population, and second on identifying common knowledge gaps within the population ^15^. In this context, several researchers have evaluated NK in adolescent athletes using a cross-sectional research design.

Previous systematic literature reviews have reported the NK of children and adolescents^16^, the NK of solely adult athletes^14, 17^ or both adolescent and adult athletes^18^. Thakur and Mathur^16^ reviewed studies investigating the relationship between NK and dietary behaviour among children and adolescents and found that higher NK was significantly associated with underweight or normal weight based on body mass index, indicating that NK may influence dietary choices. However, only two of the included studies referred to physical activity, with no description of athletic ability or comparison between physically active and inactive adolescents. Therefore, the results of this review cannot be generalized to adolescent athletic populations. A 2016 review by Trakman et al.^14^, which was subsequently updated by Janiczak et al.^17^ in 2021, excluded adolescent athletes and noted that NK was generally poor, and that several studies reported a statistically significant relationship between age and NK. Nevertheless, excluding adolescent athletes in the review leaves the conclusions unlikely to be transferrable to the adolescent athlete population^19, 20^. Heaney et al.^18^ included both adult and adolescent athletes over 13 years of age in their systematic literature review and concluded that the heterogeneity of NK assessment tools used, lack of validation methods, and unclear demographic descriptions prevented clear conclusions from being drawn on the NK level of athletes and their non-athletic comparators. Since the review by Heaney et al.^18^, a plethora of research concerning adolescent athletes’ NK levels has been conducted^9, 19, 20, 21, 22, 23, 24, 25, 26, 27, 28, 29, 30, 31, 32, 33, 34, 35, 36, 37, 38, 39, 40, 41, 42, 43, 44, 45, 46, 47, 48, 49^, presenting the need to evaluate the currently available research to determine the level of NK in adolescent athletes. This review will adapt the protocol of the previous review^18^ to target an adolescent athlete population.

## Objectives

Considering the role of nutrition in athletic performance and NK as a modifiable factor influencing food choice, and gaps in existing reviews, this review aims to:

1. Assess the level of general nutrition knowledge (GNK) and sports nutrition knowledge (SNK) among adolescent athletes between 10 and 19 years of age.
2. Identify common areas of misunderstanding within subtopics of NK, including GNK and SNK, and hydration.
3. Comparison of NK levels among adolescent athletes and non-adolescent athlete groups, including coaches, parents, adult athletes, and adolescent non-athletes

# Methods

This systematic literature review was conducted following the PRISMA guidelines^50^ (Supplementary Material - Table S1). The protocol was registered with PROSPERO (protocol registration ID CRD42022321765) and may be retrieved from <https://www.crd.york.ac.uk/prospero/display_record.php?RecordID=321765>.

## Eligibility Criteria

The World Health Organization’s definition of an adolescent (10 – 19 years of age) was employed^51^. Where the age range extended beyond 10 – 19 years, articles were included if the mean age was between 10.0 years – 19.0 years. Athletes were defined as individuals who participated in any organised sport competitively, including aesthetic sports such as gymnastics, dancing, and figure skating. All levels of sports were included; for example, recreational, competitive, high school, and elite.

The articles included in this review were original studies (cross-sectional, observational, randomised/non-randomised controlled trials, intervention), which provided a quantitative NK score in an adolescent athlete population. Studies reporting general nutrition, sports nutrition, or related domains, such as hydration and supplements, were included. Only baseline data were extracted if more than one NK assessment was conducted. NK needed to be converted into a % of correct answers and reported separately from any other assessed domain, such as attitude toward nutrition. Where studies stratified NK scores into multiple groups other than gender and sport played, the review author calculated a pooled result for ease of comparison.

Studies with or without comparison groups were included. The comparison groups included parents, coaches, adult athletes, and nonathletes of any age.

The method of questionnaire administration (e.g., handwritten, electronic, self-administered, interviewer-administered) and setting (e.g., online, at school, athletic training) were not restricted.

#### Exclusion criteria

The year of publication was restricted to 2010 onwards, as a comparable systematic review assessed the NK of athletes, including adolescents, for studies conducted before 2010^18^. Articles not published in English were excluded. Grey literature, abstracts, conference posters, editorials, and unpublished theses were excluded. Qualitative studies were excluded. Table 1 presents the inclusion and exclusion eligibility criteria of this review.

## Information Sources and Search Strategy

Searches were conducted in April 2022 by Susan Hulland (SH) using the following databases: MEDLINE, SportDiscus, SCOPUS, Web of Science, and CINAHL. In the MEDLINE search, “adolescent”, “athlete” and “nutrition knowledge” were mapped to subject headings: ADOLESCENT, ATHLETE, and NUTRITIONAL SCIENCES respectively. Keywords were then added to the search: adolescen* OR junior OR youth OR teen* OR child* OR “young adult” AND athlet* OR sport* AND “nutrition* knowledge” OR “nutrition* questionnaire” OR “nutrition* awareness”. The syntax of these terms was appropriately adjusted for each database (Supplementary Material - Table S2). The search was limited to the English language. The year of publication was not limited during the search, as articles published before 2010 were manually excluded during the screening process. This was done to assess the potential publication bias of this review by identifying articles that would otherwise be included if not for the publication date. The reference lists of the included articles were screened to identify any additional eligible articles. Where possible, “saved search alerts” were applied to retrieve articles published between the time the search was conducted and the systematic literature review was completed in May 2022.

## Study Selection

Studies retrieved from the database search were exported to Endnote for duplicate removal and subsequently exported to Covidence for eligibility screening. Two reviewers independently conducted title and abstract screening for eligibility (SH and Gina Trakman (GT) or Rebekah Alcock (RA)), with a third reviewer resolving conflicts (GT or RA). Studies that met the inclusion criteria were then screened in full text by two reviewers (SH and GT), and conflicts were resolved by discussion between the two reviewers to reach a consensus. A third reviewer (RA) resolved outstanding conflicts if consensus could not be achieved through discussion.

## Data Collection Process

A purpose-designed data extraction form based on the Academy of Nutrition and Dietetics Data Extraction Template^52^ was used to extract data from the included studies. Two reviewers (SH and RA) independently extracted data from a sample (n=5) of the included articles and reached a consensus of >90%; discrepancies were resolved by discussion, and SH conducted the remainder of the data extraction. A third reviewer (GT) checked the data extracted for inconsistencies. For each of the included articles, extracted data included author, year of publication, study information (location, study design, aim, funding sources, inclusion and exclusion criteria), demographic information of participants and comparison group where applicable (number, age, gender, type of sport played, level of sport), questionnaire information (questionnaire name and/or author, number of questions, and subsections), and outcome measures (total % score, subsection % scores where reported).

Validation of the questionnaire, as described by the author, was defined in the following categories: yes, partial, no, and unclear. Studies were rated as ‘yes’ if the authors validated the tool they used OR if they used a previously validated tool, including if any minor modifications were undertaken i.e., re-worded to apply to the local population, for example, kilojoules replacing calories. Studies were rated as ‘partial’ if they modified a previously validated questionnaire, but it was unclear if modifications were ‘minor’ or if the authors did not describe attempts to re-validate the questionnaire after modification. Studies were rated as ‘no’ if the authors used an unvalidated questionnaire or made major modifications to a previously validated questionnaire without revalidation. An ‘unclear’ rating was given if the authors did not describe validation and it was not possible to retrieve the original article to assess validation.

In cases where data were missing or there appeared to be inconsistencies, the original author was contacted for clarification and/or raw data. For inclusion in this review, the author was given a period of 14 days to respond to a request for further information.

## Quality Appraisal/Risk of Bias in Individual Studies

The quality of each included article was appraised using the Academy of Nutrition and Dietetics “Quality Criteria Checklist for Primary Research” ^52^. Two reviewers (SH and RA) independently appraised a sample of articles (n=5) that reached a consensus on >90% of the criteria. The remaining articles were appraised by a single reviewer (SH). This quality appraisal checklist was selected for use because it can be applied to all study designs included in this review, ranking an article as “positive”, “negative, or “neutral”. The checklist assesses each article based on four criteria regarding relevance and 10 validity criteria that are individually awarded “yes”, “no”, “unclear,” or “n/a”. A positive rating for the overall quality appraisal was determined by receiving “yes” for the majority of criteria, and specifically “yes” for criteria 2, 3, 6, and 7 were required when applicable.

Criteria 2 addresses selection bias in study selection. To receive a positive rating, articles were required to describe the demographic characteristics, including age, gender, and type of sport, along with a justification for the sample size used and a description of the broader relevant population. Criteria 3 was not relevant for most studies as it addresses whether study groups were comparable, as such cross-sectional studies and some intervention studies received “n/a” for criteria 3. Receiving “n/a” for this item did not hinder the potential for an overall positive rating. Criterion 6 assesses whether the intervention or procedure was discussed in detail. For this review, articles were marked as positive if they provided details of the study procedure, including ethics approval, consent obtained, and the method of administering the questionnaire. Criterion 7 refers to the validity and reliability of the measurements used, as such articles received a “yes” when they reported using a validated questionnaire or conducted measures to re-validate after modifying an existing questionnaire.

## Summary Measures

The principle outcome measure was the quantitative NK score. NK scores were retrieved as the mean correct NK knowledge score and mean correct scores for all reported subsections relating to NK, including GNK, SNK, hydration, supplements, and any other reported subsection relating to NK. All mean scores were converted to percentages to maintain consistency when discussing results. Comparisons were conducted by comparing the differences in mean scores. The secondary outcomes of identifying knowledge gaps relating to nutrition and differences between participants and comparison groups were also conducted by comparing the difference in mean scores.

## Synthesis of Results

Owing to the heterogeneity of the NK assessment tools used in the included articles, a meta-analysis and statistical measures of comparison could not be performed. As such, the results of this review will be described narratively based on the NK assessment tool used, NK domain assessed (GNK, SNK, hydration, or supplements), comparisons within studies, including the presence of a non-adolescent athlete comparison group, and the relationship between NK and age.

A narrative synthesis will also be provided regarding the quality of the included studies, focusing on criteria 2, 6, and 7 of the Academy of Nutrition and Dietetics “Quality Criteria Checklist for Primary Research” ^52^ as the primary criteria determining the quality rating.

## Risk of Bias Across Studies

Publication bias of this review due to articles not captured within the inclusion criteria was explored based on the age of participants, date of publication, and language of included articles and will be discussed qualitatively. Reporting bias was addressed by contacting authors when data were missing, or inconsistencies appeared in the reported data.

# Results

## Study Selection

The search of databases resulted in 816 articles, after the removal of duplicates (n=261) a total of 555 articles were included for the title and abstract screening. Two hundred and sixteen articles were excluded during the title and abstract screening, including 11 articles that were published before 2010. A total of 107 articles were considered eligible for full-text screening, with three articles unable to be retrieved in full text through the university document delivery portal or via contact with the study authors ^48, 53, 54^. As such, 104 full texts were screened, with 31 articles eligible for inclusion in the review. The reasons for the exclusion of articles during screening are detailed in the supplementary material (Table S3). Screening of the reference lists provided an additional three articles for review. During data extraction, two longitudinal intervention studies^55, 56^ were excluded to avoid duplicate publication bias, as their baseline data were reported in standalone articles already included in the review (Figure 1).

## Study Characteristics

Table 2 provides the results of the individual studies. Most of the studies (n=22) used a cross-sectional design. Of the remaining studies, nine investigated the impact of nutrition education in before-after studies (randomized controlled trial (n=3), quasi-experimental trial (n=6)), and one crossover trial regarding hydration status and sporting performance. Eighteen countries were represented across five continents: Europe (n=15), South America (n=5), North America (n=5), Asia (n=5), and Australia (n=2). African countries were not represented in any of the included studies.

A total of 4553 adolescent athletes were included, comprised of 1871 females, 2500 males and 182 either not reported or unable to be separated from the comparison group data. The mean age ranged from 12.7 to 18.9 years. The sample sizes ranged from 10 to 586 participants. A non-adolescent athlete comparison group of adult athletes^20, 23, 30, 33, 42^, non-athletic adults^46^, coaches^35, 40^, or non-athlete adolescents^27^ was used in nine studies and included 574 participants, comprising 205 males, 242 females and 127 whose gender was not reported. The comparison group age ranged from 16.7 to 44.3 years old. Three studies did not report the specific sports played by the study population. Eighteen studies recruited participants from one type of sport including swimming ^9, 22, 31, 49^, basketball ^28, 30, 44^, soccer ^41, 43^, gymnastics ^21^, ballet ^20^, rugby ^57^, table tennis ^23^, American football ^24^, cross-country skiing ^38^, handball ^37^, volleyball ^29^, and synchronised swimming ^40^. The remaining studies represented various sports, including track and field ^47^, aesthetic sports ^39^, endurance sports ^34, 35^, and various team and individual sports ^25, 26, 27, 36, 42, 45, 46^. Sporting levels included competitive ^9, 21, 22, 23, 26, 30, 32, 34, 35, 39, 40, 42, 49^, regional/state/national competitors ^27, 28, 29, 36, 37, 38, 43, 47^, high school/college ^19, 24, 27, 33, 41, 45, 57^, elite ^20, 44, 46^ and one study did not report sporting level ^25^. NK was assessed using 21 questionnaires across 32 studies. Twenty articles assessed a combination of two or more of the following: GNK, SNK, hydration, and supplements. The remaining articles (n=12) assessed only one aspect (GNK (n=8), SNK (n=1), and hydration (n=3)). The number of questions ranged from 10 to 113; half of the studies (n=16) used a questionnaire with 25 or fewer questions, 10 studies used more than 25 questions and four did not report the total number assessed.

## Risk of Bias Within Studies

Detailed quality assessments for all included studies, with scores assigned for each validity criterion of the Academy of Nutrition and Dietetics “Quality Criteria Checklist: Primary Research” ^52^, are available in the supplementary material (Table S4). Only two studies received an overall “positive” rating ^34, 37^, with all remaining studies (n=30) receiving “neutral” ratings, indicating a moderate quality level. The main reasons for neutral ratings were related to the selection of study subjects (criterion 2) and the use of valid and reliable measurement tools (criterion 7) followed by an inadequate description of study settings (criterion 6).

Most studies (n=28) described the demographic characteristics required by this review (age, gender and sport played); however, few (n=4) studies addressed whether the sample population was representative of the broader target population. Four studies reported the use of convenience sampling, which may not have provided a representative study population. The remaining studies often had small sample sizes, with 19 studies containing fewer than 100 participants. Of the studies with more than 100 participants (n=13), seven were recruited from a variety of sports without clarifying which population the sample should have represented. Ten studies were recruited from just one sports club, sports academy, high school, or sports team.

Validation of the questionnaire used was reported in 29 studies; most (n=13) described the tool as a “previously validated questionnaire”, and few (n=2) revalidated it after modifying the questionnaire used. Two studies^31, 41^ reported using a previously validated questionnaire, but when cross-checked with the cited article^57^ from which the questionnaire was used, it was found that the tool had not been validated, and the lack of reliability and validity testing was declared as a limitation of the study.

Most studies (n=20) provided an adequate description of the study settings and protocols. However, seven studies failed to adequately describe the method of administering the questionnaire; for example, where the questionnaire was administered, whether it was handwritten or electronic, self-administered, or interviewer administered. Five studies did not provide general details such as where the assessments took place.

## Results of Individual Studies

## Subsection Comparison

#### General Nutrition Knowledge and Sports Nutrition Knowledge

Twenty-six studies assessed GNK, including subsections on dietary recommendations, macro- and micronutrients, food choices, diet-disease relationships, and food groups/food pyramid. Sixteen studies assessed SNK; most studies (n=15) assessed SNK in conjunction with other NK domains, and only one assessed SNK as a standalone subject. The subtopics included under SNK were performance, recovery, energy/refuelling, supplements, and hydration. Four studies compared GNK to SNK, and in all cases, GNK was the highest scoring of the two sections.

Supplement knowledge was assessed as a subtopic in eight of the included studies, and no study assessed supplements as a standalone topic. Three different questionnaires were used in eight studies. Three studies^34, 35, 38^ using the Heikkila et al.^58^ questionnaire reported supplements to be the lowest scoring section. Likewise, Escribano-Ott et al.^30^ reported supplements as the lowest-scoring subsection, and Bird and Rushton ^26^ described the supplement section as the greatest source of uncertainty, with more “don’t know” responses than any other section (45%). Similarly, two studies^26, 30^ that used questionnaires based on Zinn et al.^59^ reported poor scores.

#### Hydration

Hydration knowledge was assessed in 14 studies. Eleven studies assessed hydration knowledge in combination with other NK domains, with three studies exclusively assessing hydration knowledge ^19, 28, 36^. Chia et al.^19^ created sub-sections within hydration and found that post-exercise hydration received the lowest scores (mean % correct = 33.2%), while pre- and post-exercise hydration mean % correct scores were similar at 47.2% and 47.7%, respectively.

Of the studies assessing hydration in conjunction with other NK domains, only nine reported subsection scores. Four of these studies reported hydration as the highest-scoring subsection (hydration mean score = 85.8%), three of which utilised the same questionnaire and assessed a similar population of endurance athletes in Finland (hydration mean score = 88.2%)^34, 35, 38^. Altavilla et al.^22^ reported a mean hydration score lower than the total mean score on the NK questionnaire. The remaining four studies reported hydration knowledge as average when compared to other topics.

## Comparison Across Questionnaires (Between Studies)

#### General Nutrition Knowledge Questionnaire (GNKQ)

The GNKQ was validated in an adult (non-athlete) population in the United Kingdom and contains 110 items related to dietary recommendations, sources of nutrients, choosing everyday foods, and diet-disease relationships ^60^. Four studies included in this review used this questionnaire ^20, 21, 33, 46^. Aguilo et al.^21^ made major modifications to the questionnaire, resulting in only 61 questions being included. The other three studies did not report modifications or made minor modifications.

The mean % correct scores ranged from 37.0% to 58.1% ^20, 33^. Subsection scores were reported by two studies ^33, 46^ both reported the lowest scoring section to be diet-disease relationships and the highest scores in dietary recommendations for adolescent athlete groups.

#### Leite et al., (2016)

Leite et al.^61^ validated a NK questionnaire based on previous studies ^32, 62^. The questionnaire contains 14 questions in three sections: GNK, SNK, and the Brazilian Food Pyramid (BFP). Three Brazilian studies used this tool to assess NK in adolescent athletes, none of which reported modifying the previously validated questionnaire.

The mean score of adolescent athletes ranged from 54.6%^43^ to 73.6%^42^. The highest scores in all three studies for both adolescents and adults were related to GNK, and the lowest were found in BFP. Noronha et al.^43^ did not compare the results to a non-adolescent group, while Argolo et al.^23^ and Nascimento et al.^42^ compared adolescent athletes to adult athletes who participated in the same categories of sports. Adult athletes’ mean score correct was reported as 66.7% ^23^ and 70% ^42^.

#### Walsh et al., (2011)

Walsh et al. ^57^ created a GNK questionnaire to assess NK in adolescent athletes in Ireland. The questionnaire contains 16 questions that were compiled from both validated and unvalidated questionnaires regarding hydration, supplements, energy/refuelling, and proteins. The questionnaire was pilot tested for comprehension but did not undergo further psychometric testing to determine its validity and reliability. Three studies used this questionnaire, including the author of the questionnaire ^31, 41, 57^. Manore et al.^41^ reported that the questionnaire contained 12 questions that may have been an unreported deviation from the original questionnaire or related to counting sub-questions differently than the original author.

The results showed a range of mean % correct scores from 45.6%^41^ to 68.3%^31^. Subsection scores were reported in two studies^31, 57^, which both indicated the lowest scoring section to be “protein”, and the highest “energy/refuelling”.

#### Heikkila et al. (2018)

Heikkilä et al.^58^ developed a 79-item questionnaire assessing NK topics: nutrition recommendations, supplements, hydration, recovery, and the association between food choice and body image. The questionnaire has been validated in a population of endurance athletes in Finland. All three articles that used this tool also assessed NK in Finnish athletes in various endurance sports^34, 35, 38^. Heikkilä et al.^34^ removed one question considered inappropriate for the target population, with no modifications reported in the other two studies.

The results across adolescent athletes were comparable, with total mean correct reported as 72.7% ^34^, 76% ^38^ and 77.7% ^35^. One study included an adult comparison group and reported a mean total correct of 80.8% ^35^.

#### Zinn et al. (2005)

Zinn et al. ^59^ created and validated an 84-item SNK questionnaire covering the following subtopics: nutrients, dietary reference intake (DRI), recovery, weight gain, weight loss, and supplements. Two studies utilised this questionnaire and did not report modifying it for use; however, the number of questions reported was inconsistent with the original questionnaire with Bird and Rushton ^26^ using a 90-question version and Escribano-Ott et al. ^30^ using a 23-question version, which used the subsections from the original questionnaire but reported a reduced number for each subsection. Despite the inconsistency in the number of questions reported, the total mean percentage of correct answers was comparable at 42.8% ^30^ and 43.8% ^26^.

#### Nichols et al. (2005)

Nichols et al. ^63^ created a 17-item questionnaire that was pilot-tested on college soccer players. Two studies reported the use of this questionnaire with modifications. Jusoh ^36^ reduced the total number of questions to 10 and pilot-tested the new version in schoolchildren. Carvalho et al. ^28^ used a 16-item Portuguese version of the questionnaire and reported lower internal consistency after modification.

Despite the modifications to the questionnaire, the results were somewhat comparable with Jusoh ^36^ reported mean correct scores for females and males at 73.6% and 71.8%, respectively, and Carvalho et al. ^28^ who reported mean correct scores for the total group at 79.7%.

## Nutrition Knowledge Scores of Adolescent Athletes vs Comparison Group (Within Studies)

Nine studies included a non-adolescent athlete comparison group, including adult athletes (n=4), coaches (n=2), adult non-athletes (n=1), and adolescent non-athletes (n=1).

Two studies ^23, 42^ comparing adolescent athletes with adult athletes within the same sports category used the same questionnaire^61^. Argolo et al.^23^ reported statistically significant higher overall scores in the adult comparison group (66.7% vs. 55%, p<0.5) than in the adolescent athlete group; however, the adult athletes scored lower within the GNK subsection than the adolescents. Conversely, Nascimento et al.^42^ reported lower overall scores in adult athletes (70.0%) than in adolescent athletes (73.6%). Hardy et al.^33^ compared 18–19-year-old athletes to athletes aged ≥ 20 years with similar total and subsection results (total mean correct 58.1% and 58.7%, respectively). The remaining four studies using adult comparison groups (adult athletes, adult non-athletes, and coaches) reported higher total mean correct scores in adult comparison groups.

Calella et al.^27^ compared athletes representing team sports (volleyball), aesthetic sports (gymnastics), and endurance sports (swimming) with adolescent gym members and inactive adolescents. All athlete groups scored higher on mean total knowledge scores in all subsections than non-athletes.

# Discussion

This review summarised papers on NK in adolescent athletes published since 2010. The key findings were as follows: (1) NK mean scores ranged from 33.3% to 90.6%, covering a broad range of topics related to GNK and SNK; however, the heterogeneity of the NK assessment tools used creates difficulty in making definitive statements on NK levels. (2) The areas of strength and weakness within NK topics were not consistent among studies, except where studies directly compared GNK and SNK, and it was reported that GNK scores were higher. And for supplement score, where all studies reporting a supplement sub-section reporting that scores were poor for this topic (3) The quality rating of all but two studies received a “neutral” rating with the main areas of weakness being an inadequate description of study participant selection and lack of clarity regarding the validity and reliability of data collection tools used. (4) Athletes assessed were most commonly from European countries, and multiple sporting types were often included within an individual study, of which swimming and basketball were the most reported athletes.

The NK assessment tools used varied between studies, making it difficult to directly compare the results. When the same NK assessment tool was used across multiple studies, direct comparison of results was not always possible as the individual studies often modified the questionnaire from the original form to meet the aims of the specific study, as seen in previous reviews ^14, 17^. While all NK assessment tools provided a quantitative NK score, the topics covered varied between studies. Some tools were designed to assess only a single area of NK, such as GNK ^20, 44, 49^ or hydration ^28, 36^ making comparisons between these studies illogical because of the differences in NK domains assessed. Furthermore, the level of difficulty of NK’s assessment tools has rarely been ascertained, as most studies did not provide the questionnaire used. Previous literature reviews ^14, 17, 18^ assessing the NK of athletes have similarly reported difficulty in comparing results between studies due to the heterogeneity of NK assessment tools used. It has been suggested that a greater level of insight may be obtained within individual studies using a comparison group to benchmark the results ^18^. Despite these recommendations to benchmark results, in the current review, only eight studies compared adolescent athletes to adult athletes ^20, 23, 30, 33, 42^, coaches ^35, 40^ and non-athlete adults ^46^ with only one study using an age-matched comparison group ^27^. Adult athletes scored numerically higher than adolescent athletes in all but one study^32^ that compared these groups, which is expected based on previous reports that age is associated with higher NK, likely because older individuals have higher levels of education and life experience ^23^. When an age-matched non-athlete group was used ^27^, adolescent athletes scored higher in all sections (total NK, GNK, and SNK), indicating that there may be a need for future research to include adolescent non-athlete comparison groups to gauge the level of understanding between athletes and non-athletes.

As per previous related reviews, the heterogeneity of the tools used and the subdomains of NK assessed create difficulty in identifying specific areas of strength or weakness. However, adolescent athletes consistently scored higher in the GNK than in the SNK, where the two were directly compared within studies^23, 27, 42, 43^. This finding differs from previous literature reviews, which found mixed results when measuring GNK versus SNK in adult athletes^14^. Studies that assessed and reported supplements as a subtopic indicated poor understanding, this is concerning as the use of supplements without recommendations by professionals such as accredited practicing sports dietitians or medical doctors is widespread, despite the risks of contamination with banned substances and unintended health side effects^1, 64, 65^.

Most studies were considered to have a “neutral” quality rating because of a lack of clarity regarding the study subject selection and the validation of the NK assessment tool used. Nineteen studies reported fewer than 100 participants, and rarely justified the study sample size. Furthermore, there was a lack of detail regarding the target population of the study was intending to represent. Previous reviews on adult athletes also reported limitations within studies related to inadequate statistical reporting and failure to use validated NK questionnaires^17^, with small sample sizes also common in studies on adult athletes. Of note, research among adolescents comes with obstacles that may make recruitment difficult: first, identifying a group that is large enough to provide meaningful results. This is followed by a complex process of recruiting adolescents and obtaining consent from their parent/guardian. Additionally, it is essential that researchers are sensitive to the target population and potential vulnerability; for example, the pressure to complete an NK assessment may be a concern for those at risk of disordered eating patterns due to the topic of the assessment and the known triggers of stress and negative emotions in body image dissatisfaction and disordered eating^66, 67^. Regardless of the barriers faced in recruitment, researchers must describe the method of recruitment and provide some insight into the broader community they are attempting to represent, so that the results may be interpreted accurately. A further concern regarding the quality of the articles was the lack of clarity regarding the validity of the NK assessment tools used. Several studies reported using validated questionnaires; however, upon further examination, it was determined that the questionnaire used was modified from a previously validated questionnaire without revalidation attempts, insufficiently validated, or incorrectly declared as validated. The use of a tool that has not been validated in the target population may bring into question the accuracy of the results presented.

The athletes assessed were mostly from European countries, differing from previous reviews of adult athletes ^14, 17^ which found most studies from North America primarily from the United States of America. This may be because American collegiate athletes are convenient to recruit, and thus represent a large proportion of studies on adult athletes in Western countries. Conversely, adolescent athletes are not typically grouped at the scale of collegiate athletes, and the need for parental consent to participate in research studies may prove an obstacle to recruiting large numbers. In this review, several regions were underrepresented, with Brazil being the only country included from South America, Australia the only country from Oceania, and no countries included from Africa. The lack of representation in African countries appears disproportionate when considering the number of athletes from this region; for example, endurance running is dominated by Ethiopian and Kenyan athletes^68^. The lack of funding in this region may explain the absence of studies in this region. Thirty-five different sports were reported throughout the studies; however, only one study involved many sports. Furthermore, nine studies recruited participants from at least six different sports, resulting in the total number of participants in these sports being limited, and their representation unclear. Three studies did not report the type of sports played, which brings challenges in interpreting the generalisability of the results to other adolescent athlete groups.

## Recommendations and Future Research

Implications for practice and policy

The results of this literature review indicate that there is a need for heightened education regarding sports nutrition for adolescent athletes. Considering the impact on both long-term health and athletic performance, there is great benefit to be reaped from the implementation of nutrition education programs for athletes of all levels. A systematic literature review^13^ reported the effectiveness of nutrition education interventions for athletes, however, was unable to recommended method of providing nutrition education to athletes due to poor validation of interventions and inconsistent delivery methods. However, sports nutrition education interventions for athletes may be improved by using methods such as co-design and implementing technology and social media^69^.

### Implications for Future Research

The findings from this review suggest that there is a need to improve the tools used to assess NK in adolescent athletes. Innumerable NK assessment tools are currently used worldwide, and future research would benefit from the validation of a comprehensive NK assessment tool in a range of sporting types and culturally diverse settings. The creation and use of such a tool would provide great benefit to the research community and aid the direct comparison of results between studies. Additionally, there is a lack of studies from African countries, this an area of possible future research due to the high number of sports played throughout the African continent.

## Limitations

### Limitations of Studies

The greatest limitations of the studies were small sample sizes, lack of justification for sample size or description of the target population, and the use of unvalidated or insufficiently validated tools. Several authors were contacted to clarify inconsistencies or provide missing data; however, none of the authors responded to the request for information. As such, it is possible that some outcomes or characteristics were not fully represented in this review. Few studies included comparison groups, particularly age-matched groups; therefore, it is not possible to conclude whether athletes demonstrate higher NK than groups of non-athlete adolescents. Studies rarely reported education level of the adolescents, however, this may be due to the young age of the participants restricting the education level to high school years only.

### Limitations of Review

The main limitation of this review is the implausibility of the meta-analysis owing to the heterogeneity of the NK questionnaires used throughout the studies. There was a risk of publication bias due to the exclusion of studies published before 2010, which included 11 studies that would otherwise have met the inclusion criteria. However, considering that nutrition education and assessment of NK is an evolving area of interest, it is proposed that the articles included in the current review are more likely to provide insight into the current levels of NK based on the modern understanding of nutrition requirements for adolescent athletes. Additionally, of the 11 excluded articles published before 2010 eight were included in a similar previous review ^18^. Further potential sources of bias arose in the exclusion of non-English language studies and grey literature, and the inability to source four studies for full-text screening. The review also was unable to compare the results of studies based on participant characteristics such as gender or level of sporting ability due to the heterogeneity of the studies and a lack of reporting on differences between groups based on demographic characteristics.

# Conclusion

In conclusion, this review found that adolescent athletes showed strength in GNK when compared to SNK. Adolescent athletes exhibited uncertainty regarding the use and regulation of supplements. However, other areas of strength and weakness in NK were difficult to identify owing to the heterogeneous nature of the NK assessment tools used. There is a need to consistently utilise NK tools that are validated appropriately for the target population to strengthen the results. Furthermore, the increased use of comparison groups, particularly age-matched groups, may enhance the ability to interpret results with confidence.

# Acknowledgments

This review was conducted as a part of an Honours Research year, and the authors wish to thank Dr George Moschonis for continued support and guidance throughout the literature review.

**Author contributions:**

Conception: SH and GT, design: SH and GT; literature searches: SH; independent screening: SH, GT, RA; data extraction: SH and RA; critical appraisal: SH and RA; disputes resolved by: GT or RA; preparing manuscript: SH and GT; review and editing manuscript SH, GT and RA.

**Funding:** No funding was received for this review

**Declaration of Interests:** No conflicts of interest to declare.

# Supplementary Material

Table S1: PRISMA Checklist

Table S2: Search strategies all databases

Table S3: Excluded articles in abstract and full text screening

Table S4: Critical appraisal checklist of included articles

# References

1. Desbrow B, McCormack J, Burke LM, Cox GR, Fallon K, Hislop M, et al. Sports Dietitians Australia Position Statement: Sports Nutrition for the Adolescent Athlete. International Journal of Sport Nutrition and Exercise Metabolism. 2014;24(5):570-84.10.1123/ijsnem.2014-0031

2. Fritzen AM, Lundsgaard AM, Kiens B. Dietary Fuels in Athletic Performance. Annual Review of Nutrition. 2019;39:45-73.10.1146/annurev-nutr-082018-124337

3. Norris SA, Frongillo EA, Black MM, Dong Y, Fall C, Lampl M, et al. Nutrition in adolescent growth and development. The Lancet. 2022;399(10320):172-84.10.1016/S0140-6736(21)01590-7

4. Weaver SP, Kelley L, Griggs J, Weems S, Umstattd Meyer MR. Fit and healthy family camp for engaging families in a child obesity intervention: A community health center pilot project. Family and Community Health. 2014;37(1):31-44.10.1097/FCH.0000000000000013

5. Christoph MJ, Larson NI, Winkler MR, Wall MM, Neumark-Sztainer D. Longitudinal trajectories and prevalence of meeting dietary guidelines during the transition from adolescence to young adulthood. The American Journal of Clinical Nutrition. 2019;109(3):656-64.10.1093/ajcn/nqy333

6. Thomas DT, Erdman KA, Burke LM. Position of the Academy of Nutrition and Dietetics, Dietitians of Canada, and the American College of Sports Medicine: Nutrition and Athletic Performance. Journal of the Academy of Nutrition and Dietetics. 2016;116(3):501-28.<https://doi.org/10.1016/j.jand.2015.12.006>

7. Burrows T, Harries SK, Williams RL, Lum C, Callister R. The Diet Quality of Competitive Adolescent Male Rugby Union Players with Energy Balance Estimated Using Different Physical Activity Coefficients. Nutrients. 2016;8(9):548.10.3390/nu8090548

8. Martínez-Rodríguez A, Martínez-Olcina M, Hernández-García M, Rubio-Arias JÁ, Sánchez-Sánchez J, Lara-Cobos D, et al. Mediterranean Diet Adherence, Body Composition and Performance in Beach Handball Players: A Cross Sectional Study. International Journal of Environmental Research and Public Health. 2021;18(6):2837.10.3390/ijerph18062837

9. Philippou E, Middleton N, Pistos C, Andreou E, Petrou M. The impact of nutrition education on nutrition knowledge and adherence to the Mediterranean Diet in adolescent competitive swimmers. Journal of Science and Medicine in Sport. 2017;20(4):328-32.10.1016/j.jsams.2016.08.023

10. Zanella PB, August PM, Alves FD, Matté C, de Souza CG. Association of Healthy Eating Index and oxidative stress in adolescent volleyball athletes and non-athletes. Nutrition. 2019;60:230-4.<https://doi.org/10.1016/j.nut.2018.10.017>

11. Birkenhead KL, Slater G. A Review of Factors Influencing Athletes’ Food Choices. Sports Medicine. 2015;45(11):1511-22.10.1007/s40279-015-0372-1

12. Trakman GL. The development and validation of the nutrition for sport knowledge questionnaire (NSKQ) and abridged nutrition for sport knowledge questionnaire (A-NSKQ) to investigate the sports nutrition knowledge of Australian athletes. [Doctoral Thesis]. La Trobe University Research Online.: La Trobe University; 2018.

13. Tam R, Beck KL, Manore MM, Gifford J, Flood VM, O'Connor H. Effectiveness of Education Interventions Designed to Improve Nutrition Knowledge in Athletes: A Systematic Review. Sports Medicine. 2019;49(11):1769-86.10.1007/s40279-019-01157-y

14. Trakman G, Forsyth A, Devlin B, Belski R. A Systematic Review of Athletes’ and Coaches’ Nutrition Knowledge and Reflections on the Quality of Current Nutrition Knowledge Measures. Nutrients. 2016;8(9):570.10.3390/nu8090570

15. Devlin BL, Belski R. Exploring General and Sports Nutrition and Food Knowledge in Elite Male Australian Athletes. International Journal of Sport Nutrition and Exercise Metabolism. 2015;25(3):225-32.<https://dx.doi.org/10.1123/ijsnem.2013-0259>

16. Thakur S, Mathur P. Nutrition knowledge and its relation with dietary behaviour in children and adolescents: a systematic review. International Journal of Adolescent Medicine and Health. 2021.doi:10.1515/ijamh-2020-0192

17. Janiczak A, Devlin BL, Forsyth A, Trakman GL. A systematic review update of athletes’ nutrition knowledge and association with dietary intake. British Journal of Nutrition. 2022;128((6) ):1156-69.10.1017/s0007114521004311

18. Heaney S, O’Connor H, Michael S, Gifford J, Naughton G. Nutrition Knowledge in Athletes: A Systematic Review. International Journal of Sport Nutrition and Exercise Metabolism. 2011;21(3):248-61.10.1123/ijsnem.21.3.248

19. Chia M, Mukherjee S, Huang D. Thirst for Drink Knowledge: How Singaporean Youth Athletes Measure Up in an Exercise Hydration Knowledge Questionnaire. International Journal of Sports Science & Coaching. 2015;10(5):841-50.10.1260/1747-9541.10.5.841

20. Wyon MA, Hutchings KM, Wells A, Nevill AM. Body Mass Index, Nutritional Knowledge, and Eating Behaviors in Elite Student and Professional Ballet Dancers. Clinical Journal of Sport Medicine. 2014;24(5):390-6.10.1097/JSM.0000000000000054

21. Aguilo A, Lozano L, Tauler P, Nafria M, Colom M, Martinez S. Nutritional Status and Implementation of a Nutritional Education Program in Young Female Artistic Gymnasts. Nutrients. 2021;13(5).10.3390/nu13051399

22. Altavilla C, Prats-Moya MS, Perez PC. Hydration and nutrition knowledge in adolescent swimmers. Does water intake affect urine hydration markers after swimming? International Journal of Applied Exercise Physiology. 2017;6(4):37-45.10.22631/ijaep.v6i4.220

23. Argôlo D, Borges J, Cavalcante A, Silva G, Maia S, Ramos A, et al. Poor dietary intake and low nutritional knowledge in adolescent and adult competitive athletes: a warning to table tennis players. Nutricion Hospitalaria. 2018;35(5):1124-30.10.20960/nh.1793

24. Atkins WC, McDermott BP, Koji K, Adams JD, Kavouras SA. Effects of Hydration Educational Intervention in High School Football Players. Journal of Strength & Conditioning Research (Lippincott Williams & Wilkins). 2021;35(2):385-90.10.1519/jsc.0000000000003866

25. Bakhtiar M, Masud-ur-Rahman M, Kamruzzaman M, Sultana N, Rahman SS. Determinants of nutrition knowledge, attitude and practices of adolescent sports trainee: A cross-sectional study in Bangladesh. Heliyon. 2021;7(4).10.1016/j.heliyon.2021.e06637

26. Bird SP, Rushton BD. Nutritional knowledge of youth academy athletes. BMC Nutrition. 2020;6(1).10.1186/s40795-020-00360-9

27. Calella P, Gallè F, Di Onofrio V, Buono P, Liguori G, Valerio G. Gym Members Show Lower Nutrition Knowledge than Youth Engaged in Competitive Sports. Journal of the American College of Nutrition. 2021;40(5):465-71.10.1080/07315724.2020.1792375

28. Carvalho P, Oliveira B, Barros R, Padräo P, Moreira P, Teixeira VH. Impact of Fluid Restriction and ad Libitum Water Intake or an 8% Carbohydrate-Electrolyte Beverage on Skill Performance of Elite Adolescent Basketball Players. International Journal of Sport Nutrition and Exercise Metabolism. 2011;21(3):214-21.10.1123/ijsnem.21.3.214

29. Daniel NVS, Jurgensen LP, Padovani RD, Juzwiak CR. Impact of an Interdisciplinary Food, Nutrition and Health Education Program for adolescent Brazilian volleyball players. Revista De Nutricao-Brazilian Journal of Nutrition. 2016;29(4):567-77.10.1590/1678-98652016000400011

30. Escribano-Ott I, Mielgo-Ayuso J, Calleja-González J. A Glimpse of the Sports Nutrition Awareness in Spanish Basketball Players. Nutrients. 2021;14(1):27.10.3390/nu14010027

31. Foo WL, Faghy MA, Sparks A, Newbury JW, Gough LA. The Effects of a Nutrition Education Intervention on Sports Nutrition Knowledge during a Competitive Season in Highly Trained Adolescent Swimmers. Nutrients. 2021;13(8):2713.10.3390/nu13082713

32. Gonçalves CB, Nogueira JAD, Da Costa THM. The Food Pyramid Adapted to Physically Active Adolescents as a nutrition education tool. Revista Brasileira de Ciencias do Esporte. 2014;36(1):29-44.10.1590/S0101-32892014000100003

33. Hardy R, Kliemann N, Evansen T, Brand J. Relationship Between Energy Drink Consumption and Nutrition Knowledge in Student-Athletes. Journal of Nutrition Education and Behavior. 2017;49(1):19-26.e1.<https://dx.doi.org/10.1016/j.jneb.2016.08.008>

34. Heikkilä M, Lehtovirta M, Autio O, Fogelholm M, Valve R. The Impact of Nutrition Education Intervention with and Without a Mobile Phone Application on Nutrition Knowledge Among Young Endurance Athletes. Nutrients. 2019;11(9):2249.10.3390/nu11092249

35. Heikkilä M, Valve R, Lehtovirta M, Fogelholm M. Nutrition Knowledge Among Young Finnish Endurance Athletes and Their Coaches. International Journal of Sport Nutrition and Exercise Metabolism. 2018;28(5):522-7.10.1123/ijsnem.2017-0264

36. Jusoh N. Relationship between hydration status, hydration knowledge and fluid intake behaviour among school athletes of selected Perak sport schools. Journal of Sports Science and Physical Education. 2014:11-9

37. Jusoh N, Lee JLF, Tengah RY, Azmi SH, Suherman A. Association between nutrition knowledge and nutrition practice among Malaysian adolescent handball athletes. Malaysian Journal of Nutrition. 2021;27(2):279-91.10.31246/MJN-2020-0113

38. Kettunen O, Heikkilä M, Linnamo V, Ihalainen JK. Nutrition Knowledge Is Associated with Energy Availability and Carbohydrate Intake in Young Female Cross-Country Skiers. Nutrients. 2021;13(6).10.3390/nu13061769

39. Laramée C, Drapeau V, Valois P, Goulet C, Jacob R, Provencher V, Lamarche B. Evaluation of a Theory-Based Intervention Aimed at Reducing Intention to Use Restrictive Dietary Behaviors Among Adolescent Female Athletes. Journal of Nutrition Education and Behavior. 2017;49(6):497-504.e1.10.1016/j.jneb.2017.03.009

40. Mandic GF, Peric M, Krzelj L, Stankovic S, Zenic N. Sports Nutrition and Doping Factors in Synchronized Swimming: Parallel Analysis among Athletes and Coaches. Journal of Sports Science & Medicine. 2013;12(4):753-60

41. Manore MM, Patton-Lopez MM, Meng Y, Wong SS. Sport Nutrition Knowledge, Behaviors and Beliefs of High School Soccer Players. Nutrients. 2017;9(4).10.3390/nu9040350

42. Nascimento M, Silva D, Ribeiro S, Nunes M, Almeida M, Mendes-Netto R. Effect of a Nutritional Intervention in Athlete's Body Composition, Eating Behaviour and Nutritional Knowledge: A Comparison between Adults and Adolescents. Nutrients. 2016;8(9):535.10.3390/nu8090535

43. Noronha DC, Santos M, Santos AA, Corrente LGA, Fernandes RKN, Barreto ACA, et al. Nutrition Knowledge is Correlated with a Better Dietary Intake in Adolescent Soccer Players: A Cross-Sectional Study. Journal of Nutrition and Metabolism. 2020;2020.10.1155/2020/3519781

44. Sánchez-Díaz S, Yanci J, Raya-González J, Scanlan AT, Castillo D. A Comparison in Physical Fitness Attributes, Physical Activity Behaviors, Nutritional Habits, and Nutritional Knowledge Between Elite Male and Female Youth Basketball Players. Frontiers in Psychology. 2021;12.10.3389/fpsyg.2021.685203

45. Saribay AK, Kirbaş Ş. Determination of nutrition knowledge of adolescents engaged in sports. Universal Journal of Educational Research. 2019;7(1):40-7.10.13189/ujer.2019.070106

46. Spendlove JK, Heaney SE, Gifford JA, Prvan T, Denyer GS, O'Connor HT. Evaluation of general nutrition knowledge in elite Australian athletes. British Journal of Nutrition. 2012;107(12):1871-80.10.1017/S0007114511005125

47. Supriya V. R, L. Knowledge, attitude and dietary practices of track and field athletic men and women aged 18-22. International Journal of Innovative Research & Development. 2013;2(11):399-404

48. Walsh AM, Madigan S, Cleary J, Corish C. The nutrition knowledge and weight-making practices of elite and non-elite Irish youth boxers. Proceedings of the Nutrition Society. 2013;72(OCE3):E179-E.10.1017/s0029665113002024

49. Webb MC, Beckford SE. Nutritional knowledge and attitudes of adolescent swimmers in Trinidad and Tobago. Journal of Nutrition and Metabolism. 2014;2014.10.1155/2014/506434

50. Page MJ, McKenzie JE, Bossuyt PM, Boutron I, Hoffmann TC, Mulrow CD, et al. The PRISMA 2020 statement: an updated guideline for reporting systematic reviews. BMJ. 2021;372:n71.10.1136/bmj.n71

51. Singh JA, Siddiqi M, Parameshwar P, Chandra-Mouli V. World Health Organization Guidance on Ethical Considerations in Planning and Reviewing Research Studies on Sexual and Reproductive Health in Adolescents. Journal of Adolescent Health. 2019;64(4):427-9.10.1016/j.jadohealth.2019.01.008

52. Academy of Nutrition and Dietetics. Evidence Analysis Manual: Steps in the Academy Evidence Analysis Proccess 2016. Available from: <https://www.andeal.org/vault/2440/web/files/2016_April_EA_Manual.pdf>.

53. Bilgic P, Tamer F, Yabanci N, Hamamcilar O, Bilgic SC, Hongu N. Exploring the use of text messages for increasing nutrition knowledge and improving dietary practices in teen athletes. FASEB Journal. 2014;28(1)

54. Carter K, Nash K, Walden A, Roberts A. Does The Source Of Nutrition Information Affect Nutrition Knowledge. Medicine and Science in Sports and Exercise. 2021;53:298-

55. Spronk I, Heaney SE, Prvan T, O'Connor HT. Relationship Between General Nutrition Knowledge and Dietary Quality in Elite Athletes. International Journal of Sport Nutrition and Exercise Metabolism. 2015;25(3):243-51.10.1123/ijsnem.2014-0034

56. Patton-Lopez MM, Manore MM, Branscum A, Meng Y, Wong SS. Changes in sport nutrition knowledge, attitudes/beliefs and behaviors following a two-year sport nutrition education and life-skills intervention among high school soccer players. Nutrients. 2018;10(11).10.3390/nu10111636

57. Walsh M, Cartwright L, Corish C, Sugrue S, Wood-Martin R. The Body Composition, Nutritional Knowledge, Attitudes, Behaviors, and Future Education Needs of Senior Schoolboy Rugby Players in Ireland. International Journal of Sport Nutrition and Exercise Metabolism. 2011;21(5):365-76

58. Heikkila M, Valve R, Lehtovirta M, Fogelholm M. Development of a nutrition knowledge questionnaire for young endurance athletes and their coaches. Scandinavian Journal of Medicine and Science in Sports. 2018;28(3):873-80.<https://dx.doi.org/10.1111/sms.12987>

59. Zinn C, Schofield G, Wall C. Development of a psychometrically valid and reliable sports nutrition knowledge questionnaire. Journal of Science and Medicine in Sport. 2005;8(3):346-51.<https://doi.org/10.1016/S1440-2440(05)80045-3>

60. Parmenter K, Wardle J. Development of a general nutrition knowledge questionnaire for adults. European Journal of Clinical Nutrition. 1999;53(4):298-308.10.1038/sj.ejcn.1600726

61. Leite MDM, Santos Barbosa Machado AC, Da Silva DG, Falcão Raposo OF, Mendes Netto RS. Conocimiento sobre alimentacion y nutricion despues del desarrollo de actividades de educacion alimentaria entre ninos y adolescentes deportistas Pensar a Prática. 2016;19(1).10.5216/rpp.v19i1.40619

62. Zawila LG, Steib CM, Hoogenboom B. The female collegiate cross-country runner: nutritional knowledge and attitudes. Journal of Athletic Training (National Athletic Trainers' Association). 2003;38(1):67-74

63. Nichols PE, Jonnalagadda SS, Rosenbloom CA, Trinkaus M. Knowledge, Attitudes, and Behaviors Regarding Hydration and Fluid Replacement of Collegiate Athletes. International Journal of Sport Nutrition and Exercise Metabolism. 2005;15(5):515

64. Mettler S, Lehner G, Morgan G. Widespread Supplement Intake and Use of Poor Quality Information in Elite Adolescent Swiss Athletes. International Journal of Sport Nutrition and Exercise Metabolism. 2022;32(1):41-8.10.1123/ijsnem.2021-0043

65. Whitehouse G, Lawlis T. Protein supplements and adolescent athletes: A pilot study investigating the risk knowledge, motivations and prevalence of use. Nutrition &amp; Dietetics. 2017;74(5):509-15.10.1111/1747-0080.12367

66. Wells KR, Jeacocke NA, Appaneal R, Smith HD, Vlahovich N, Burke LM, Hughes D. The Australian Institute of Sport (AIS) and National Eating Disorders Collaboration (NEDC) position statement on disordered eating in high performance sport. British Journal of Sports Medicine. 2020;54(21):1247-58.10.1136/bjsports-2019-101813

67. Naumann E, Svaldi, J., Wyschka, T., Heinrichs, M., von Dawans, B. Stress-induced body dissatisfaction in women with binge eating disorder. Journal of Abnormal Psychology. 2018;127(6):548-58.10.1037/abn0000371

68. Zani ALS, Gouveia MH, Aquino MM, Quevedo R, Menezes RL, Rotimi C, et al. Genetic differentiation in East African ethnicities and its relationship with endurance running success. PloS One. 2022;17(5):e0265625.10.1371/journal.pone.0265625

69. Scharoun L, Mews G. Designed by kids for kids: Design strategies for improved outcomes for children’s health and wellbeing in suburban environments. AIP Conference Proceedings. 2020;2230(1).10.1063/5.0002311

70. Decher NR, Casa DJ, Yeargin SW, Ganio MS, Levreault ML, Dann CL, et al. Hydration status, knowledge, and behavior in youths at summer sports camps. International Journal of Sports Physiology and Performance. 2008;3(3):262-78.10.1123/ijspp.3.3.262

71. Calella P, Iacullo VM, Valerio G. Validation of a General and Sport Nutrition Knowledge Questionnaire in Adolescents and Young Adults: GeSNK. Nutrients. 2017;9(5):439.10.3390/nu9050439

72. Jürgensen LP, Daniel NVS, Padovani RDC, Lourenço LCDA, Juzwiak CR. Avaliação da qualidade da dieta de atletas de esportes coletivos. Revista Brasileira de Cineantropometria e Desempenho Humano. 2015;17(3):280.10.5007/1980-0037.2015v17n3p280

73. Lima EDS, Monteiro EADA, Andrade APD. Educação nutricional na escola do primeiro grau em Pernambuco (Brasil): diagnóstico. Revista de Saúde Publica. 1985;19(6):508-20.10.1590/s0034-89101985000600003

74. Triches RM, Giugliani ERJ. Obesidade, práticas alimentares e conhecimentos de nutrição em escolares. Revista de Saúde Publica. 2005;39(4):541-7.10.1590/s0034-89102005000400004

75. Sedek R, Yih TY. Dietary Habits and Nutrition Knowledge among Athletes and Non-Athletes in National University of Malaysia (UKM). Pakistan journal of nutrition : PJN. 2014;13(12):752-9.10.3923/pjn.2014.752.759

76. Morissette É, Laramée C, Drapeau V, Couture S, Valois P, Goulet C, et al. Determinants of Restrictive Dietary Behaviors among Female High School Athletes. Health Behavior and Policy Review. 2015;2(5):378-87.10.14485/hbpr.2.5.6

77. Kondric M, Sekulic D, Uljevic O, Gabrilo G, Zvan M. Sport nutrition and doping in tennis: an analysis of athletes' attitudes and knowledge. Journal of Sports Science & Medicine. 2013;12(2):290-7

78. Turconi G, Celsa M, Rezzani C, Biino G, Sartirana MA, Roggi C. Reliability of a dietary questionnaire on food habits, eating behaviour and nutritional knowledge of adolescents. European Journal of Clinical Nutrition. 2003;57(6):753-63.10.1038/sj.ejcn.1601607

79. Öz F, Aydin R, Önsuz M, METİNTAŞ S, Emiral G. Development of a reliable and valid adolescence nutritional knowledge questionnaire. Progress in Nutrition. 2016;18(2)

80. Ozdoğan Y, Ozcelik AO. Evaluation of the nutrition knowledge of sports department students of universities. Journal of the International Society of Sports Nutrition. 2011;8(1):11.10.1186/1550-2783-8-11

# Table and Figure Legend

Table 1: Eligibility Criteria

Table 2: Summary of Results

Figure 1. PRISMA flow diagram of the inclusion eligibility screening process

## Table 1. Eligibility criteria

| **Included** | **Excluded** |
| --- | --- |
| Original research, study designs included: cross-sectional, randomised/non-randomised controlled trial, observational, intervention | Abstracts, conference posters, editorials, letters to the editor, literature reviews, and unpublished theses |
| Adolescent athletes (10-19 years) of any sport type and level | Mean age beyond the range of 10 – 19, all non-athletes |
| English language | Non-English language |
| Quantitative nutrition knowledge score including GNK, SNK, and related sub-domains | Qualitative studies, or studies that did not provide quantitative NK score |
| Published between 2010 – April 2022 | Published before 2010 |

| Table 2. Summary of Results | | | |  |  |  |  |  |  |  |  |  |  |  |
| --- | --- | --- | --- | --- | --- | --- | --- | --- | --- | --- | --- | --- | --- | --- |
| **Author, Year, Country** | **Study Design** | **Sport played; *sport level*** | **Adolescent athletes (N, gender)** | **Adolescent athlete’s age (mean ± SD, or range if mean NR)** | **Non-adolescent athlete comparison (N, gender)** | **Non-adolescent athlete comparison group age (mean ± SD, or range if mean NR)** | **Questionnaire used, number of questions** | **Validation** | **Topic and subsections (if reported)** | **Adolescent athletes mean correct total (%) ± SD** | **Adolescent athletes mean correct subsection (%) ± SD** | **Comparison group Mean correct total (%) ± SD** | **Comparison group Mean correct subsection (%) ± SD** | **Quality rating** |
| **Aguilo et al.^21^, (2021): Spain** | Quasi-experimental | Gymnastics; *competitive* | *n*=24, F:24 | 14.1 ± 2.3 | n/a | n/a | Adapted from GNKQ Parmenter and Wardle ^60^, (1999); *n*=61 | Partial | GNK | **45.9 ± 2.79*** | NR | n/a | n/a | Neutral |
| **Altavilla et al.^22^, (2017): Spain** | Cross-sectional | Swimming; *competitive* | *n*=86, F:46 M:40 | 11-16 | n/a | n/a | Adapted from Zawila et al.^62^, (2003); *n*=NR | Unclear | SNK  Hydration (4) | 33.3 ± 12.6 | Hydration: 28.6 ±28.6 | n/a | n/a | Neutral |
| **Argolo et al.^23^, (2018); Brazil** | Cross-sectional | Table tennis; *competitive* | *n*=25, F:2 M:23 | *n*=13.8 ± 2.5 | *Adult athletes: n*=17 M:17 | 33.6 ±10.8 | Leite et al. ^61^. (2016); *n*=14 | Yes | GNK (3) Brazilian food pyramid (BFP) (1) SNK (10) | 55 ± 12 | GNK: 82.5 ± 23^#^  BFP: 14 ± 8 SNK: 63.2 ± 20 | 66.7 ± 10 | GNK: 97 ± 9.4^#^  BFP: 17.6 ± 10  SNK: 79.4 ± 21^#^ | Neutral |
| **Atkins et al. ^24^, (2021); USA** | Non-randomised controlled trial | American football; *high school* | Group 1: *n=21, M:21* Group 2*: n*=20, M:20 | Group 1: *16 ± 1* Group 2*:* 16 ± 1 | n/a | n/a | Modified Decher et al. ^70^, (2008); *n*=25 | No | GNK Hydration | Group 1: 90 ± 6 Group 2: 89 ± 9 | NR | n/a | n/a | Neutral |
| **Bakhtiar et al.^25^, (2021); Bangladesh** | Cross-sectional | Cricket, football, athletics, basketball, volleyball, archery, table tennis, karate, wushu, taekwondo, hockey;  *Level NR* | *n*=260, F:27 M:233 | 15.5 ± 1.83 | n/a | n/a | Author designed; *n*=22 | Yes | GNK | **58.0 ± 9.9*** | NR | n/a | n/a | Neutral |
| **Bird and Rushton^26^, (2020); Australia** | Cross-sectional | Netball, officiating, basketball, softball, hockey, tennis, lawn bowls and triathlon;  *Competitive* | *n=*101, F: 64 M: 37 | 15.3 ±1.4 | n/a | n/a | Zinn et al. ^59^, (2005); *n*=90 | Yes | Nutrients, dietary reference intake (DRI), Recovery, weight gain, weight loss, supplements | 43.8 ± 11.4 | Nutrients: 51, DRI: 27, hydration: 46, recovery: 54, Weight gain: 3-78, weight loss: 56, supplements: NR | n/a | n/a | Neutral |
| **Author, Year, Country** | **Study Design** | **Sport played; *sport level*** | **Adolescent athletes (N, gender)** | **Adolescent athletes age (mean ± SD, or range if mean NR)** | **Non-adolescent athlete comparison (N, gender)** | **Non-adolescent athlete comparison group age (mean ± SD, or range if mean NR)** | **Questionnaire used, number of questions** | **Validation** | **Topic and subsections (if reported)** | **Adolescent athletes mean correct total (%) ± SD** | **Adolescent athletes mean correct subsection (%) ± SD** | **Comparison group Mean correct total (%) ± SD** | **Comparison group Mean correct subsection (%) ± SD** | **Quality rating** |
| **Calella et al.^27^, (2021); Italy** | Cross-sectional | Gym members, volleyball, swimmer, gymnasts; *regional /national competitors* | Total *n=*131, ***Volleyball:*** *n*=43, F:21, M:22 ***Swimmers:*** *n*=39, F:19, M: 20 ***Gymnasts:*** *n*=49, F:49 | ***Volleyball:*** 17.7 ±1.5 ***Swimmers:*** 16.6±1.8  ***Gymnasts:*** 16.2±1.8 | Total *n* = 80, ***Gym members:*** *n*=33, F:16 M:17, ***Inactive youth:*** *n* = 47, F:28 M: 19 | ***Gym members:*** 16.7 ±1.1 ***Inactive youth:*** 16.7 ± 1.5 | GeSNK, Calella et al.^71^, (2017); *n*=62 | Yes | GNK (29), SNK (33) | Total: Volleyball: 60.9 ± 12.3Swimmers: 62.4 ± 7.9Gymnasts: 66.6 ± 11.5 | GNK – Volleyball: 63.6 ± 13.8 Swimmers: 63.0 ± 10.3 Gymnasts: 68.4 ± 12.5 SNK - Volleyball: 55.8 ± 13.3 Swimmers: 63.0 ± 10.9 Gymnasts: 63.0 ± 13.3 | Total: Gym members: 49.6 ± 14.3Inactive youth: 53.2 ± 8.7 | GNK -Gym members: 50.6 ± 15.8 Inactive youth: 55.5 ± 10.9 SNK - Gym members: 47.6 ± 15.8 Inactive youth: 48.8 ± 13.0 | Neutral |
| **Carvalho et al. ^28^, (2011); Portugal** | Cross-over trial | Basketball; *national competitors* | *n=*12, M:12 | 14.8 ± 0.45 | n/a | n/a | Modified Nichols et al.^63^ , (2005); *n*=16 hydration | Partial | Hydration | 79.7 ± 14.6 | n/a | n/a | n/a | Neutral |
| **Chia et al. ^19^, (2015); Singapore** | Cross-sectional | NR; *high school* | *n*=586, F: 259 M: 322 Other:5 | 13.9 ± 2.5 | n/a | n/a | Author designed; *n* =30 | Yes | Hydration:  Pre-exercise hydration (9), during exercise hydration (13), post-exercise hydration (8) | 44.1 ± 14.0 | Pre-exercise: 47.2± 22.0 During exercise: 48.7 ± 16.9 Post-exercise: 33.2 ±17.9 | n/a | n/a | Neutral |
| **Daniel et al. ^29^, (2016); Brazil** | Intervention | Volleyball; *state competitors* | *n*=10, F:10 | 17.2 ± 0.94 | n/a | n/a | Jurgensen et al. ^72^, (2015); *n*= NR | Yes | Macro nutrients, micronutrients, food groups, sport nutrition beliefs | 57.0 ± 9.9 | Macro nutrients: 72.0 ±16.6, micronutrients: 27.0 ±22.6, food groups: 98.9 ±3.5^#^, sport nutrition beliefs 70.9 ± 15.9 | n/a | n/a | Neutral |
| **Author, Year, Country** | **Study Design** | **Sport played; *sport level*** | **Adolescent athletes (N, gender)** | **Adolescent athletes age (mean ± SD, or range if mean NR)** | **Non-adolescent athlete comparison (N, gender)** | **Non-adolescent athlete comparison (mean ± SD, or range if mean NR)** | **Questionnaire used, number of questions** | **Validation** | **Topic and subsections (if reported)** | **Adolescent athletes mean correct total (%) ± SD** | **Adolescent athletes mean correct subsection (%) ± SD** | **Comparison group Mean correct total (%) ± SD** | **Comparison group Mean correct subsection (%) ± SD** | **Quality rating** |
| **Escribano-Ott et al. ^30^(2021); Spain** | Cross-sectional | Basketball; *competitive* | *n*=69, F:37 M:32 | 15-18 | *Adult athletes*  *Non-pro: n=14, F:8 M:6 Pro:* n=21, F:10 M:11 | *Non-pro: NR Pro: NR* | Zinn et al. ^59^, (2005); *n*=23 | Partial | Nutrients, hydration, recovery, weight management, supplements | **42.8*** | Nutrients: 51.5 ± 16.1, hydration: 47.2 ± 18.8, recovery: 41.9 ± 1.71, mass management: 47.7 ± 10.06, supplementation: 25.7 ± 22.5 | **Non-pro: 49.9***  **Pro: 46.0*** | Non-pro: Nutrients: 59.5 ± 11.7, hydration: 57.9 ± 13.9, recovery: 55.2 ± 14.9, mass management: 51.4 ± 7.1, supplementation: 25.3 ± 19.9Pro: Nutrients: 51.2 ± 18.4, hydration: 51.8 ± 24.7, recovery: 45.0 ± 20.4, mass management: 47.9 ± 17.3, supplementation: 33.8 ± 27.3 | Neutral |
| **Foo et al. ^31^, (2021); United Kingdom** | Quasi-experimental | Swimming; *competitive* | *n*=15, F:10 M:5 | 15.5 ± 1.1 | n/a | n/a | Walsh et al. ^57^, (2011); n=16 | No | Energy and refuelling, hydration, supplements, protein | 68.3 | Energy and refuelling: 81.0, hydration: 68.9, supplements: 66.7, protein: 40.0 | n/a | n/a | Neutral |
| **Gonçalves et al. ^32^, (2014); Brazil** | Intervention | NR; *competitive* | *n*=58, F:27 M:31 | 13.7 ± 0.77 | n/a | n/a | Modified Lima et al. ^73^, (1985). Triches & Giugliani ^74^, (2005); *n*= NR | Unclear | GNK, food pyramid | 59.9 ± 18 | NR | n/a | n/a | Neutral |
| **Hardy et al.^33^, (2017); United States of America** | Cross-sectional | NR; *College* | *n*=95, NR | 18 -19 | *n* = 99, NR | >20 | Modified GNKQ, Parmenter and Wardle ^60^, (1999); *n*=NR | Partial | Dietary recommendations, nutrients, food choice, diet-disease relationships | **58.1 ± 0.9*** | ***Dietary recommendations: 62.7 ± 0.9, nutrients: 60.0 ± 0.9, food choice: 57.1 ± 1.9, diet-disease relationships: 47.1± 1.6** | **58.7 ± 0.9*** | ***Dietary recommendation: 65.5 ± 0.9, nutrients: 60.2 ± 1.0, food choice: 61.4 ± 1.7, diet-disease relationships: 47.1± 1.9** | Neutral |
| **Author, Year, Country** | **Study Design** | **Sport played; *sport level*** | **Adolescent athletes (N, gender)** | **Adolescent athletes age (mean ± SD, or range if mean NR)** | **Non-adolescent athlete comparison (N, gender)** | **Non-adolescent athlete comparison (mean ± SD, or range if mean NR)** | **Questionnaire used, number of questions** | **Validation** | **Topic and subsections (if reported)** | **Adolescent athletes mean correct total (%) ± SD** | **Adolescent athletes mean correct subsection (%) ± SD** | **Comparison group Mean correct total (%) ± SD** | **Comparison group Mean correct subsection (%) ± SD** | **Quality rating** |
| **Heikkilä et al. ^35^, (2018); Finland** | Cross-sectional | Cross-country skiing, orienteering, biathlon, running/racewalking, triathlon, swimming/rowing/canoeing, athletics, cycling; *competitive* | *n*=312, F:156 M:156 | 17.9 ± 1.2 | *Coaches: n*=94, F:25 M:69 | 44.3 ± 12.3 | Heikkilä et al. ^58^, (2018); *n*=79 | Yes | Nutrition recommendations, supplements, hydration, recovery, food choice/body image | 72.7 ± 8.8 | Nutrition recommendations: 69.2 ± 10.4, supplements: 66.0 ± 22.6, hydration: 85.2 ± 15.3, recovery: 72.8 ± 13.0, food choice/body image: 84.0 ± 14.7 | 80.8 ± 8.7 | Nutrition recommendations: 76.8 ± 10.8, supplements: 79.8 ± 20.0, hydration: 90.9 ± 10.9^#^, recovery: 82.0 ± 11.8, food choice/body image: 91.4 ± 9.9^#^ | Neutral |
| **Heikkilä et al. ^34^, (2019); Finland** | Randomised controlled trial | Cross-country skiing, orienteering, biathlon, running/race walking, triathlon; *competitive* | Group 1: *n*=37, F:18 M:19 Group 2: *n*=42, F:17 M:25 | Group 1: 18.0 ± 1.4  Group 2: 18.0 ± 1.4 | n/a | n/a | Heikkilä et al. ^58^, (2018); *n*=78 | Yes | Nutrition recommendations, supplements, hydration, recovery, food choice/body image | Total participants: 77.7 ± 7.6 | ***Group 1:*** Nutrition recommendations: 75.8, supplements: 71.4, hydration: 87.2, recovery: 77.3, food choice/body image: 83 ***Group 2:*** Nutrition recommendations: 75.2, supplements: 73.5, hydration: 87.8, recovery: 77.9, food choice/body image: 87.9 | n/a | n/a | Positive |
| **Jusoh ^36^, (2014); Malaysia** | Cross-sectional | Football, athletics, Sepak takraw, hockey, rugby, netball; *regional/state/national competitors* | *n*=70, F:25 M:45 | 14.0 ± 1 | n/a | n/a | Modified Nichols et al. ^63^, (2005); *n*=10 | Partial | Hydration | F:73.6 ± 6.3  M:71.8 ± 11.7 | NR | n/a | n/a | Neutral |
| **Author, Year, Country** | **Study Design** | **Sport played; *sport level*** | **Adolescent athletes (N, gender)** | **Adolescent athletes age (mean ± SD, or range if mean NR)** | **Non-adolescent athlete comparison (N, gender)** | **Non-adolescent athlete comparison (mean ± SD, or range if mean NR)** | **Questionnaire used, number of questions** | **Validation** | **Topic and subsections (if reported)** | **Adolescent athletes mean correct total (%) ± SD** | **Adolescent athletes mean correct subsection (%) ± SD** | **Comparison group Mean correct total (%) ± SD** | **Comparison group Mean correct subsection (%) ± SD** | **Quality rating** |
| **Jusoh et al. ^37^, (2021); Malaysia** | Cross-sectional | Handball; *national competitors* | *n*=312, F: 157 M:155 | 16 ± 1 | n/a | n/a | Modified Razalee and Tan ^75^, (2014); *n*=11 | Yes | GNK, SNK | F: 78.6 ± 13.1 M: 77.3 ± 12.1 | n/a | n/a | n/a | Positive |
| **Kettunen et al. ^38^ (2021); Finland** | Cross-sectional | Cross-country skiers; *national competitors* | *n*=19, F:19 | 16.7 ± 0.7 | n/a | n/a | Heikkilä et al. ^58^, (2018); *n*=79 | Yes | Nutrition recommendations, supplements, hydration, recovery, food choice/body image | 76.0 ± 7.3 | Nutrition recommendations: 73.4 ± 8.4, supplements: 67.4 ± 19.1, hydration: 92.5 ± 10.0, recovery: 71.8 ± 14.6, food choice/body image: 87.1 ± 11.3 | n/a | n/a | Neutral |
| **Laramée et al. ^39^ (2017); Canada** | Randomised controlled trial | Various aesthetic sports: synchronised swimming, gymnastics, dancing, cheerleading; *competitive* | Group 1: *n*=37, F:37 Group 2: *n*=33, F:33 | Group 1: 14.1 ± 1.5 Group 2: 13.1 ± 1.2 | n/a | n/a | Morisette et al. ^76^, (2015); *n*=37 | Yes | Carbohydrates (8), proteins (9), lipids (8), SNK (7) | Group 1: 60.8 Group 2: 51.4 | NR | n/a | n/a | Neutral |
| **Mandic et al. ^40^, (2013); Croatia/Serbia** | Cross-sectional | Synchronised swimming; *competitive* | *n* = 82, NR | 17 ± 1.92 | *Coaches:*  *n*=28, NR | 30 ± 5.26 | Kondric et al. ^77^, (2013); *n*=18 | Yes | SNK | **30.9 ± 16.1** | NR | **45.2 ± 17.3** | n/a | Neutral |
| **Manore et al.^41^, (2017); United States of America** | Cross-sectional | Soccer; *high school* | *n*=535, F:297 M:238 | 15.3 ± 1.14 | n/a | n/a | Walsh et al. ^57^, (2011); n=12 | No | Hydration, protein/carbohydrate, supplements, food choice | 45.6 | NR | n/a | n/a | Neutral |
| **Author, Year, Country** | **Study Design** | **Sport played; *sport level*** | **Adolescent athletes (N, gender)** | **Adolescent athletes age (mean ± SD, or range if mean NR)** | **Non-adolescent athlete comparison (N, gender)** | **Non-adolescent athlete comparison (mean ± SD, or range if mean NR)** | **Questionnaire used, number of questions** | **Validation** | **Topic and subsections (if reported)** | **Adolescent athletes mean correct total (%) ± SD** | **Adolescent athletes mean correct subsection (%) ± SD** | **Comparison group Mean correct total (%) ± SD** | **Comparison group Mean correct subsection (%) ± SD** | **Quality rating** |
| **Nascimento et al. ^42^, (2016); Brazil** | Quasi-experimental | Fighting, athletics, cycling, swimming, tennis, beach volleyball, surfing, rowing, sailing; *competitive* | *n*=21, F:6 M:15 | 15.4 | *Adult athletes: n*=11, M:11 | 23.7 | Leite et al. ^61^, (2016); *n*=14 | Yes | GNK (3), BFP (1), SNK (10) | 73.6 | GNK: 92, BFP: 37, SNK: 83.3 | 70.0 | GNK: 89.2, BFP: 28.4, SNK: 84.5 | Neutral |
| **Noronha et al. ^43^, (2020); Brazil** | Cross-sectional | Soccer; *state competitors* | *n*=73, M:73 | 17.0 ± 1.3 | n/a | n/a | Leite et al. ^61^, (2016); n=14 | Yes | GNK (3), BFP (1), SNK (10) | 54.6 ± 13.6 | GNK: 75.7 ± 22.6 BFP: 16.3 ± 9.2 SNK: 67.8 ± 21.1 | n/a | n/a | Neutral |
| **Philippou et al. ^9^, (2017); Cyprus** | Intervention | Swimming; *competitive* | *n*=34, F:11 M:23 | 15.2 ± 1.5 | n/a | n/a | Author designed; *n*=10 | Yes | Macronutrients/micronutrients (9), Mediterranean diet (1) | 70.0 | NR | n/a | n/a | Neutral |
| **Sanchez-Diaz et al. ^44^, (2021); Spain** | Cross-sectional | Basketball; *elite* | *n*=23, F:10 M:13 | F: 12.7 ± 0.5 M:13.5 ± 0.3 | n/a | n/a | Modified Turconi et al. ^78^,  (2003); *n*=11 | Yes | GNK | 42.0 | NR | n/a | n/a | Neutral |
| **Saribay and Kirbay ^45^, (2019); Turkey** | Cross-sectional | Football, basketball, volleyball, athletics, handball, other; *high school/ college* | *n*=495, F:100 M:395 | 14-19 | n/a | n/a | Öz et al. ^79^, (2016); *n*=38 | Yes | Dietary recommendations (9), food items (21), diet-disease relationship (8) | **F: 53.8  M: 52.6** | NR | n/a | n/a | Neutral |
| **Spendlove et al. ^46^, (2012); Australia** | Cross-sectional | Life-saving, rugby league, other (NR); *elite* | *n*=175, F:99 M:76 | 18.9 ± 4.9 | *Community* (CM): *n*=116, F:84 M:32 *Dietetic trained (DT):* *n*=53, F:46 M:7 | CM: 21.9 ± 4.2  DT: 29.1 ± 8.5 | Modified GNKQ, Parmenter and Wardle ^60^, (1999); *n*=45 | Yes | Dietary recommendations, nutrients, food choice, diet-disease relationship | 57.6 | Dietary recommendations; 65.4, nutrients: 60.9, food choice: 60.0, diet-disease relationship 45.0 | CM: 63.1 DT: 86.2 | **CM**: Dietary recommendations65.4, nutrients 67.7, food choice 64.0, diet-disease relationship 50.0 **DT**: Dietary recommendations77.7, nutrients 92.0, food choice 76.0, diet-disease relationship 83.5 | Neutral |
| **Author, Year, Country** | **Study Design** | **Sport played; *sport level*** | **Adolescent athletes (N, gender)** | **Adolescent athletes age (mean ± SD, or range if mean NR)** | **Non-adolescent athlete comparison (N, gender)** | **Non-adolescent athlete comparison (N, gender)** | **Questionnaire used, number of questions** | **Validation** | **Topic and subsections (if reported)** | **Adolescent athletes mean correct total (%) ± SD** | **Adolescent athletes mean correct subsection (%) ± SD** | **Comparison group Mean correct total (%) ± SD** | **Comparison group Mean correct subsection (%) ± SD** | **Quality rating** |
| **Supriya & Ramaswami,^47^, (2013); India** | Cross-sectional | Track and field; *state/ national competitors* | *n*=178, F:107 M:71 | 18.0 ± 3.2 | n/a | n/a | Author designed; *n*=10 | Partial | GNK, performance, food groups, carbohydrates, proteins, antioxidants, sports drinks, hydration | 52.6 ± 15 | GNK 92, performance: 39 food groups: 42, carbohydrate: 22, proteins: 81, antioxidants: 58, sports drink: 26, hydration: 42 | n/a | n/a | Neutral |
| **Walsh et al. ^57^, (2011); Ireland** | Cross-sectional | Rugby; *high school* | *n*=203, M:203 | 16-18 | n/a | n/a | Author designed; *n*=16 | No | Hydration, supplements, energy/ refuelling, protein | 59.6 ± 12.8 | Hydration: 76.4 ± 20.7, supplements 69.3 ± 28.6, energy/ refuelling 57.1 ± 15.4, protein 39.2 ± 31.4 | n/a | n/a | Neutral |
| **Webb and Beckford ^49^, (2014); Trinidad and Tobago** | Cross-sectional | Swimming; *competitive* | *n*=220, F:98 M:122 | 14.6 ± 2.5 | n/a | n/a | Adapted Ozdogan and Ozcelik ^80^, (2011); Zawila et al. ^62^, (2003); *n*= 21 | Partial | GNK | **52.2 ± 13.8*** | NR | n/a | n/a | Neutral |
| **Wyon et al.^20^ (2014); United Kingdom** | Cross-sectional | Ballet; *elite* | *n*=139, F:86 M:53 | 11-18 | Professional ballet dancers *n*=41, F:25 M:16 | 19-39 | GNKQ, Parmenter and Wardle ^60^, (1999); NR | Partial | GNK | **F: 37.1*+ M:37.0*+** | NR | F:51.0 M:54.0 | NR | Neutral |

M: Male, F: Female N: number; SD: standard deviation GNKQ: General Nutrition Knowledge Questionnaire; GNK: General Nutrition Knowledge; SNK: Sports Nutrition Knowledge; BFP: Brazilian food pyramid; NR:not reported; GeSNK: General and Sports Nutrition Knowledge; DRI: Dietary Reference Intake.

#: SD reported as crossing 100%, reported as stated by original author

***Review author calculated mean total % based on mean score reported.**

***+: Author calculated pooled results due to stratification by multiple factors**
