# Supplementary material for: Adolescent athletes have better general than sports nutrition knowledge and lack awareness of supplement recommendations: a systematic literature review
Source: Br J Nutr. 2023 Dec 6;131(8):1362–76. doi: 10.1017/S0007114523002799 (PMC10950453; doi:10.1017/S0007114523002799)
Supplement: Hulland et al. supplementary material 3 — Hulland et al. supplementary material [file S0007114523002799sup003.docx]

## Table S2 – Search Strategies All Databases

| **CINAHL** | | | **OVID Medline** | | **SCOPUS** | **SPORTDISCUS** | | | **Web of Science** | |
| --- | --- | --- | --- | --- | --- | --- | --- | --- | --- | --- |
| S1 | (MH "Health Knowledge") | **Expanders** - Apply equivalent subjects  **Search modes** - Boolean/Phrase | 1 | exp Adolescent/ | ( ( TITLE-ABS-KEY ( "nutrition* awareness" ) ) | S1 | athlete* | **Search modes** - Boolean/Phrase | 1 | **ALL=("young adult")** |
| S2 | "nutrition* knowledge" |  | 2 | adolescen*.mp. | OR | S2 | sport* |  | 2 | **ALL=(child*)** |
| S3 | "nutrition* questionnaire" |  | 3 | junior.mp. | ( TITLE-ABS-KEY ( "nutrition* questionnaire" ) ) | S3 | S1 OR S2 |  | 3 | **ALL=(teen*)** |
| S4 | "nutrition* awareness" |  | 4 | youth.mp. | OR | S4 | adolescen* |  | 4 | **ALL=(youth)** |
| S5 | (MH "Athletes+") |  | 5 | teen*.mp. | ( TITLE-ABS-KEY ( "nutrition* knowledge" ) ) ) | S5 | youth |  | 5 | **ALL=(junior)** |
| S6 | athlet* |  | 6 | child*.mp. | AND | S6 | junior |  | 6 | **ALL=(adolescen*)** |
| S7 | sport* |  | 7 | “young adult”.mp. | ( ( TITLE-ABS-KEY ( sport* ) ) | S7 | teen* |  | 7 | **#1 OR #2 OR #3 OR #4 OR #5 OR #6** |
| S8 | S5 OR S6 OR S7 |  | 8 | 1 or 2 or 3 or 4 or 5 or 6 or 7 | OR | S8 | Child* |  | 8 | **ALL=(sport*)** |
| S9 | (MH "Adolescence+") |  | 9 | exp Athletes/ | ( TITLE-ABS-KEY ( athlet* ) ) ) | S9 | S4 OR S5 OR S6 OR S7 OR S8 |  | 9 | **ALL=(athlet*)** |
| S10 | adolescen* |  | 10 | athlet*.mp. | AND | S10 | "nutrition* knowledge" |  | 10 | **#8 OR #9** |
| S11 | youth |  | 11 | sport*.mp. | ( ( TITLE-ABS-KEY ( adolescen* ) ) | S11 | "nutrition* questionnaire" |  | 11 | **ALL=("nutrition* awareness")** |
| S12 | teen* |  | 12 | 9 or 10 or 11 | OR | S12 | “nutrition* awareness” |  | 12 | **ALL=("nutrition* questionnaire")** |
| S13 | child* |  | 13 | exp Nutritional Sciences/ | ( TITLE-ABS-KEY ( junior ) ) | S13 | S10 OR S11 OR S12 |  | 13 | **ALL=("nutrition* knowledge")** |
| S14 | "young adult" |  | 14 | "nutrition* knowledge".mp. | OR | S14 | S3 AND S9 AND S13 |  | 14 | **#11 OR #12 OR #13** |
| S15 | S9 OR S10 OR S11 OR S12 OR S13 OR S14 |  | 15 | "nutrition* questionnaire".mp. | ( TITLE-ABS-KEY ( youth ) ) | S15 | S3 AND S9 AND S13 | **Narrow by Language:**- English  **Search modes** - Boolean/Phrase | 15 | **#7 AND #10 AND #14** |
| S16 | S1 OR S2 OR S3 OR S4 |  | 16 | "nutrition* awareness".mp. | OR |  |  |  |  |  |
| S17 | S8 AND S15 AND S16 |  | 17 | 13 or 14 or 15 or 16 | ( TITLE-ABS-KEY ( teen* ) ) |  |  |  |  |  |
| S18 | S8 AND S15 AND S16 | **Limiters** - English Language  **Expanders** - Apply equivalent subjects  **Search modes** - Boolean/Phrase | 18 | 8 and 12 and 17 | OR |  |  |  |  |  |
|  |  |  | 19 | limit 18 to English language | ( TITLE-ABS-KEY ( child* ) ) |  |  |  |  |  |
|  |  |  |  |  | OR |  |  |  |  |  |
|  |  |  |  |  | ( TITLE-ABS-KEY ( "young adult" ) ) ) |  |  |  |  |  |
|  |  |  |  |  | AND |  |  |  |  |  |
|  |  |  |  |  | ( LIMIT-TO ( LANGUAGE ,  "English" ) ) |  |  |  |  |  |
