# Supplementary material for: Adolescent athletes have better general than sports nutrition knowledge and lack awareness of supplement recommendations: a systematic literature review
Source: Br J Nutr. 2023 Dec 6;131(8):1362–76. doi: 10.1017/S0007114523002799 (PMC10950453; doi:10.1017/S0007114523002799)
Supplement: Hulland et al. supplementary material 4 — Hulland et al. supplementary material [file S0007114523002799sup004.docx]

## Table S4 – Critical Appraisal Breakdown of Criteria

| Name | Q1 | Q2 | Q3 | Q4 | Q5 | Q6 | Q7 | Q8 | Q9 | Q10 | Overall rating |
| --- | --- | --- | --- | --- | --- | --- | --- | --- | --- | --- | --- |
| Aguilo et al., 2021 | Yes | Unclear | n/a | Unclear | n/a | Unclear | Unclear | Yes | Unclear | Yes | Neutral (3) |
| Altavilla et al., 2017 | Yes | Unclear | n/a | Unclear | n/a | Yes | Unclear | Yes | Yes | Yes | Neutral (5) |
| Argolo, et al., 2018 | Yes | Unclear | n/a | n/a | n/a | Yes | Yes | Yes | Yes | Yes | Neutral (6) |
| Atkins et al., 2021 | Yes | Unclear | Yes | Unclear | n/a | Yes | Unclear | Yes | Yes | Unclear | Neutral (5) |
| Bakhtiar et al., 2021 | Yes | Unclear | n/a | n/a | n/a | Yes | Unclear | Yes | Yes | Yes | Neutral (5) |
| Bird et al., 2020 | Yes | Unclear | n/a | n/a | n/a | Yes | Unclear | Yes | Yes | Yes | Neutral (5) |
| Calella et al., 2021 | Yes | Unclear | Unclear | n/a | n/a | Yes | Yes | Yes | Yes | Yes | Neutral (6) |
| Carvahlo et al., 2011 | Yes | Unclear | n/a | Unclear | Unclear | Yes | Unclear | Yes | Yes | Unclear | Neutral (4) |
| Chia et al., 2016 | Yes | Unclear | n/a | n/a | n/a | Yes | Yes | Yes | Yes | Unclear | Neutral (5) |
| Daniel et al., 2016 | Yes | Unclear | n/a | Yes | n/a | Unclear | Yes | Yes | Unclear | Unclear | Neutral (4) |
| Escribano-Ott et al, 2022 | Yes | No | n/a | n/a | n/a | Unclear | Unclear | Yes | Yes | Yes | Neutral (4) |
| Foo et al., 2021 | Yes | No | n/a | Unclear | n/a | Yes | Unclear | Yes | Yes | Yes | Neutral (5) |
| Gonçalves et al., 2014 | Yes | No | Yes | Yes | n/a | Unclear | Unclear | Yes | Yes | Unclear | Neutral (5) |
| Hardy et al., 2017 | Yes | No | n/a | n/a | n/a | Yes | Yes | Yes | Yes | Unclear | Neutral (5) |
| Heikkilä et al., 2018 | Yes | Unclear | Yes | Yes | n/a | Yes | Yes | Yes | Yes | Unclear | Neutral (7) |
| Heikkilä et al., 2019 | Yes | Yes | Yes | Yes | n/a | Yes | Yes | Yes | Yes | Unclear | Positive (8) |
| Jusoh, 2014 | Yes | Unclear | n/a | n/a | n/a | Unclear | Unclear | Yes | Unclear | Unclear | Neutral (2) |
| Jusoh et al., 2021 | Yes | Yes | n/a | n/a | n/a | Yes | Yes | Yes | Yes | Yes | Positive (7) |
| Kettunen et al., 2021 | Yes | Unclear | n/a | n/a | n/a | Yes | Yes | Yes | Yes | Yes | Neutral (6) |
| Laramée et al., 2017 | Yes | No | Unclear | No | n/a | Yes | Yes | Yes | Yes | Yes | Neutral (6) |
| Mandic et al., 2013 | Yes | Unclear | n/a | n/a | n/a | Yes | Yes | Yes | Yes | Unclear | Neutral (5) |
| Manore et al., 2017 | Yes | Yes | n/a | n/a | n/a | Yes | Unclear | Yes | Yes | Yes | Neutral (6) |
| Nascimento et al., 2016 | Yes | Unclear | n/a | n/a | n/a | Unclear | Yes | Yes | Unclear | Yes | Neutral (4) |
| Noronha et al., 2020 | Yes | Unclear | n/a | n/a | n/a | Yes | Yes | Yes | Yes | Unclear | Neutral (5) |
| Philippou et al., 2017 | Yes | Unclear | n/a | n/a | n/a | Unclear | Unclear | Yes | Yes | Unclear | Neutral (3) |
| Sanchez-Diaz et al., 2021 | Yes | Unclear | n/a | n/a | n/a | Yes | Unclear | Yes | Yes | Unclear | Neutral (4) |
| Saribay & Kirbay, 2019 | Yes | Unclear | n/a | n/a | n/a | Unclear | Yes | Unclear | Unclear | Unclear | Neutral (2) |
| Spendlove et al., 2012 | Yes | Unclear | n/a | n/a | n/a | Yes | Yes | Yes | Yes | Yes | Neutral (6) |
| Supriya & Ramaswami, 2013 | Yes | Unclear | n/a | n/a | n/a | Unclear | Unclear | Yes | No | Unclear | Neutral (2) |
| Walsh et al., 2011 | Yes | Unclear | n/a | n/a | n/a | Unclear | No | Yes | Yes | Unclear | Neutral (3) |
| Webb et al., 2014 | Yes | Yes/unclear | n/a | n/a | n/a | Unclear | Unclear | Yes | Yes | Unclear | Neutral (3) |
| Wyon et al., 2014 | Yes | Unclear | n/a | n/a | n/a | Yes | Yes | Yes | Unclear | Unclear | Neutral (4) |
