# Supplementary material for: Adolescent athletes have better general than sports nutrition knowledge and lack awareness of supplement recommendations: a systematic literature review
Source: Br J Nutr. 2023 Dec 6;131(8):1362–76. doi: 10.1017/S0007114523002799 (PMC10950453; doi:10.1017/S0007114523002799)
Supplement: Hulland et al. supplementary material 5 — Hulland et al. supplementary material [file S0007114523002799sup005.docx]

## Table S3 - Articles Excluded in Abstract and Full Text Screening

| Number | Reference | Reason for exclusion |
| --- | --- | --- |
| 1 | Abbasi, I. S., Lopez, R. M., Kuo, Y.-T., & Shapiro, B. S. (2021). Efficacy of an Educational Intervention for Improving the Hydration Status of Female Collegiate Indoor-Sport Athletes. *Journal of Athletic Training (Allen Press)*, *56*(8), 829-835. <https://doi.org/10.4085/1062-6050-0495.19> | Non-adolescent |
| 2 | Abbey, E. L., Wright, C. J., & Kirkpatrick, C. M. (2017). Nutrition practices and knowledge among NCAA Division III football players. *Journal of the International Society of Sports Nutrition*, *14*(1). <https://doi.org/10.1186/s12970-017-0170-2> | Non-adolescent |
| 3 | Alaunyte, I., Perry, J. L., & Aubrey, T. (2015). Nutritional knowledge and eating habits of professional rugby league players: Does knowledge translate into practice? *Journal of the International Society of Sports Nutrition*, *12*(1). <https://doi.org/10.1186/s12970-015-0082-y> | Non-adolescent |
| 4 | Ali, A., Al-Siyabi, M. S., Waly, M. I., & Kilani, H. A. (2015). Assessment of nutritional knowledge, dietary habits and nutrient intake of university student athletes. *Pakistan Journal of Nutrition*, *14*(5), 293-299. <https://doi.org/10.3923/pjn.2015.293.299> | Non-adolescent |
| 5 | Bidil, S., & Kaya, M. (2021). Analysis of the attitude of badminton athletes about healthy nutrition/ Badminton sporcularinin sağlikli beslenmeye ilişkin tutum düzeyinin incelenmesi. *Journal of Physical Education & Sports Science / Beden Egitimi ve Spor Bilimleri Dergisi*, *15*(2), 201-213. | No quantitative NK score |
| 6 | Chalcarz, W., Radzimirska-Graczyk, M., & Janczewski, M. J. (2004). General nutritional knowledge in children and adolescents practicing fencing. *New Medicine*, *7*(1), 8-11. | Mean NK score not reported |
| 7 | Challis, J., Cahalan, R., Jakeman, P., NiBhriain, O., Cronin, L., & Reeves, S. (2020). Dietary Intake, Body Composition, and Nutrition Knowledge of Irish Dancers. *Journal of Dance Medicine & Science*, *24*(3), 105-112. <https://doi.org/10.12678/1089-313X.24.3.105> | Non-adolescent |
| 8 | Chen, S. L., Zhu, X. H., Welk, G. J., Kim, Y., Lee, J. M., & Meier, N. F. (2014). Using Sensewear armband and diet journal to promote adolescents' energy balance knowledge and motivation. *Journal of Sport and Health Science*, *3*(4), 326-332. <https://doi.org/10.1016/j.jshs.2013.07.003> | Non-athlete |
| 9 | Chiang, L.M., Shamsuddin, A.F., & Mahmood, T.M.T. (2018). Knowledge, attitude, and practice on doping of Malaysian student athletes. *Asian Journal of Pharmaceutical and Clinical Research, 11*(5), 72-76. | No quantitative NK score |
| 10 | Coccia, C., Fernandes, S. M., & Altiti, J. (2020). Tweeting for Nutrition: Feasibility and Efficacy Outcomes of a 6-Week Social Media-Based Nutrition Education Intervention for Student-Athletes. *Journal of Strength & Conditioning Research (Lippincott Williams & Wilkins)*, *34*(7), 2084-2092. <https://doi.org/10.1519/JSC.0000000000002500> | Non-adolescent |
| 11 | Condo, D., Lohman, R., Kelly, M., & Carr, A. (2019). Nutritional Intake, Sports Nutrition Knowledge and Energy Availability in Female Australian Rules Football Players. *Nutrients*, *11*(5), 971-971. <https://doi.org/10.3390/nu11050971> | Non-adolescent |
| 12 | Curtis, C., Russell, M., & Ranchordas, M. K. (2018). Enhancing dietary practices, general nutrition knowledge and body composition of a female International Rugby Union player incorporating smartphone application technology. *Journal of Sports Medicine & Physical Fitness*, *58*(3), 366-368. <https://doi.org/10.23736/S0022-4707.17.07623-X> | Wrong study design (letter to the editor) |
| 13 | Deguchi, M., Yokoyama, H., Hongu, N., Watanabe, H., Ogita, A., Imai, D., Suzuki, Y., & Okazaki, K. (2021). Eating perception, nutrition knowledge and body image among para-athletes: Practical challenges in nutritional support. *Nutrients*, *13*(9). <https://doi.org/10.3390/nu13093120> | Non-adolescent |
| 14 | Demirozu, B. E., Pehlivan, A., & Camliguney, A. F., (2012). Nutrition knowledge and behaviours of children aged 8-12 who attend sport schools. *Procedia Social and Behavioural Sciences*, *46*, 4713-4717. <https://doi.org/10.1016/j.sbspro.2012.06.324> | No quantitative NK score |
| 15 | Devlin, B. L., Leveritt, M. D., Kingsley, M., & Belski, R. (2017). Dietary Intake, Body Composition, and Nutrition Knowledge of Australian Football and Soccer Players: Implications for Sports Nutrition Professionals in Practice. *International Journal of Sport Nutrition & Exercise Metabolism*, *27*(2), 130-138. <https://doi.org/10.1123/ijsnem.2016-0191> | Non-adolescent |
| 16 | Doering, T. M., Reaburn, P. R., Cox, G., & Jenkins, D. G. (2016). Comparison of Postexercise Nutrition Knowledge and Postexercise Carbohydrate and Protein Intake Between Australian Masters and Younger Triathletes. *International Journal of Sport Nutrition & Exercise Metabolism*, *26*(4), 338-346. https://doi.org/10.1123/ijsnem.2015-0289 | Non-adolescent |
| 17 | Doyle-Lucas, A. F., & Davy, B. M. (2011). Development and Evaluation of an Educational Intervention Program for Pre-professional Adolescent Ballet Dancers: Nutrition for Optimal Performance. *Journal of Dance Medicine & Science*, *15*(2), 65-75. | No quantitative NK score |
| 18 | Duarte, M. A. D., Trakman, G. L., Mello, J. B., de Andrade, M. X., Carlet, R., Machado, C. L. F., Pinto, R. S., & Voser, R. D. (2021). Nutritional Knowledge and Eating Habits of the National Brazilian Futsal Team. *Revista Espanola De Nutricion Humana Y Dietetica*, *25*. <https://doi.org/10.14306/renhyd.25.S1.1393> | Non-adolescent |
| 19 | Dunn, D., Turner, L. W., & Denny, G. (2007). Nutrition knowledge and attitudes of college athletes. *Sport Journal*, *10*(4), 10p-10p. | Non-adolescent |
| 20 | Elsahoryi, N. A., Trakman, G., & Al Kilani, A. (2021). General and sports nutrition knowledge among Jordanian adult coaches and athletes: A cross-sectional survey. *PLoS ONE [Electronic Resource]*, *16*(11), e0258123. <https://doi.org/10.1371/journal.pone.0258123> | Non-adolescent |
| 21 | EskİCİ, G., & Ersoy, G. (2016). An evaluation of wheelchair basketball players' nutritional status and nutritional knowledge levels. *Journal of Sports Medicine & Physical Fitness*, *56*(3), 259-268. | Non-adolescent |
| 22 | Folasire, O. F., Akomolafe, A. A., & Sanusi, R. A. (2015). Does Nutrition Knowledge and Practice of Athletes Translate to Enhanced Athletic Performance? Cross-Sectional Study Amongst Nigerian Undergraduate Athletes. *Global Journal of Health Science*, *7*(5), 215-225. <https://dx.doi.org/10.5539/gjhs.v7n5p215> | Non-adolescent |
| 23 | Heaney, S., O'Connor, H., Michael, S., Gifford, J., & Naughton, G. (2011). Nutrition Knowledge in Athletes: A Systematic Review. *International Journal of Sport Nutrition & Exercise Metabolism*, *21*(3), 248-261. <https://doi.org/10.1123/ijsnem.21.3.248> | Wrong study design (systematic literature review) |
| 24 | Heydenreich, J., Carlsohn, A., Mayer, F., & Heydenreich, J. (2014). Nutrition knowledge and food choice in young athletes. *International Journal of Pediatric Research.* | Age not stated |
| 25 | Horvath, G., Meyer, N. L., Konrad, M., & Muller, E. (2014). Determination of Nutrition Knowledge of Young Athletes in Austria Translation and Adaptation of the "Adolescent Sport Nutrition Knowledge Questionnaire". *Ernahrungs Umschau*, *61*(9), M476-M481 | Wrong study design (adaptation of questionnaire to different language) |
| 26 | Jacobson, B. H., Sobonya, C., & Ransone, J. (2001). Nutrition practices and knowledge of college varsity athletes: a follow-up. *Journal of Strength & Conditioning Research*, *15*(1), 63-68. | Age not stated |
| 27 | Jagim, A. R., Fields, J. B., Magee, M., Kerksick, C., Luedke, J., Erickson, J., & Jones, M. T. (2021). The Influence of Sport Nutrition Knowledge on Body Composition and Perceptions of Dietary Requirements in Collegiate Athletes. *Nutrients*, *13*(7), 2239-2239. <https://doi.org/10.3390/nu13072239> | Non-adolescent |
| 28 | Jessri, M., RashidKhani, B., & Zinn, C. (2010). Evaluation of Iranian college athletes' sport nutrition knowledge. *International Journal of Sport Nutrition & Exercise Metabolism*, *20*(3), 257-263. <https://doi.org/10.1123/ijsnem.20.3.257> | Non-adolescent |
| 29 | Jovaní Casano, C., Martínez Costa, C., & Gómez Cabrera, M. C. (2011). Nutritional assessment of school age children and adolescents who are high-level cycling competitors. Dietetics recommendations for the athletic child. *Acta Pediatrica Espanola*, *69*(9), 385-391. | Non-English |
| 30 | Klein, D. J., Eck, K. M., Walker, A. J., Pellegrino, J. K., & Freidenreich, D. J. (2021). Assessment of sport nutrition knowledge, dietary practices, and sources of nutrition information in ncaa division iii collegiate athletes. *Nutrients*, *13*(9). <https://doi.org/10.3390/nu13092962> | Non-adolescent |
| 31 | Kose, G., Sam, C. T., Mizrak, O., Acar, H., & Tutkun, E. (2021). Nutrition and dehydration: Players should learn how to bring them to life. *Progress in Nutrition*, *23*(1). <https://doi.org/10.23751/pn.v23i1.9448> | Non-adolescent |
| 32 | Krick, R., Brown, K., Ramsay, S., & Brown, A. F. (2017). Changes in Knowledge of the Female Athlete Triad among Female High School Athletes Following a Brief Nutrition Education Intervention. *Journal of the Academy of Nutrition & Dietetics*, *117*(10), A142-A142. <https://doi.org/10.1016/j.jand.2017.08.085> | Grey literature |
| 33 | Lohman, R., Carr, A., & Condo, D. (2019). Nutritional Intake in Australian Football Players: Sports Nutrition Knowledge and Macronutrient and Micronutrient Intake. *International Journal of Sport Nutrition & Exercise Metabolism*, *29*(3), 289-296. [https://dx.doi.org/10.1123/ijsnem.2018-0031](https://doi.org/https://dx.doi.org/10.1123/ijsnem.2018-0031) | Non-adolescent |
| 34 | Loosli, A. R., Benson, J., Gillien, D. M., & Bourdet, K. (1986). Nutrition habits and knowledge in competitive adolescent female gymnasts. / Habitudes alimentaires des adolescents gymnastes de competition. *Physician & Sportsmedicine*, *14*(8), 118-120;129. | Mean NK score not reported |
| 35 | Lopez, M. M., Marchesano, A. C., & Navarro, F. (2010). Nutritional evaluation of aerobic gymnastic and artistic gymnastic athletes. *Rbne-Revista Brasileira De Nutricao Esportiva*, *4*(23), 365-370. | Non-English |
| 36 | Magee, P. J., Gallagher, A. M., & McCormack, J. M. (2017). High Prevalence of Dehydration and Inadequate Nutritional Knowledge Among University and Club Level Athletes. *International Journal of Sport Nutrition & Exercise Metabolism*, *27*(2), 158-168. [https://dx.doi.org/10.1123/ijsnem.2016-0053](https://doi.org/https://dx.doi.org/10.1123/ijsnem.2016-0053) | Age not stated |
| 37 | Marchi, A., Onida, M., Lanfranconi, M., Scaringi, M., Gualea, M., Lameri, E., De Nigris, V. M., Zin, C., & Tenconi, T. (2001). Evaluation of nutritional knowledge in adolescent athletes and in their trainers. *Minerva pediatrica*, *53*(5), 505-506. | Non-English |
| 38 | Miran, K., Damir, S., Ognjen, U., Goran, G., & Milan, Z. (2013). Sport Nutrition and Doping in Tennis: An Analysis of Athletes' Attitudes and Knowledge. *Journal of Sports Science & Medicine*, *12*(2), 290-297. | Non-adolescent |
| 39 | Nascimento, M., Silva, D., Ribeiro, S., Nunes, M., Almeida, M., & Mendes-Netto, R. (2016). Effect of a Nutritional Intervention in Athlete's Body Composition, Eating Behaviour and Nutritional Knowledge: A Comparison between Adults and Adolescents. *Nutrients*, *8*(9), 535. <https://doi.org/10.3390/nu8090535> | Non-adolescent |
| 40 | Nor Azizam, N. S., Yusof, S. N., Amon, J. J., Ahmad, A., Safii, N. S., & Jamil, N. A. (2022). Sports Nutrition and Food Knowledge among Malaysian University Athletes. *Nutrients*, *14*(3), 572. <https://doi.org/10.3390/nu14030572> | Non-adolescent |
| 41 | Ocana, M., Folle, R., & Saldana, C. (2009). Habits and nutrition knowledge of adolescent performance swimmers. *European Journal of Human Movement*, *23*, 95-106. | Non-English |
| 42 | Partida, S., Marshall, A., Henry, R., Townsend, J., & Toy, A. (2018). Attitudes toward Nutrition and Dietary Habits and Effectiveness of Nutrition Education in Active Adolescents in a Private School Setting: A Pilot Study. *Nutrients*, *10*(9), 1260. <https://doi.org/10.3390/nu10091260> | Non-athlete |
| 43 | Patton-Lopez, M. M., Manore, M. M., Branscum, A., Meng, Y., & Wong, S. S. (2018). Changes in sport nutrition knowledge, attitudes/beliefs and behaviors following a two-year sport nutrition education and life-skills intervention among high school soccer players. *Nutrients*, *10*(11). <https://doi.org/10.3390/nu10111636> | Duplicate data reported in another article |
| 44 | Rash, C. L., Malinauskas, B. M., Duffrin, M. W., Barber-Heidal, K., & Overton, R. F. (2008). Nutrition-related knowledge, attitude, and dietary intake of college track athletes. *Sport Journal*, *11*(1), 1-8. | Non-adolescent |
| 45 | Rastmanesh, R., Taleban, F. A., Kimiagar, M., & Mehrabi, Y. (2007). Nutritional knowledge and attitudes in athletes with physical disabilities. *Journal of Athletic Training*, *42*(1), 99-105. | Non-adolescent |
| 46 | Reeves, S., & Foote, A. (2003). The influence of nutritional knowledge and residential status on the diets of professional youth soccer players. *Journal of Sports Sciences*, *21*(4), 332-333. | Non-adolescent |
| 47 | Renard, M., Anton-Solanas, A., Kelly, D. T., & Ó Catháin, C. (2022). Evaluation of nutrition knowledge in elite and sub-elite Gaelic football players. *Science and Medicine in Football*, *6*(1), 82-88. <https://doi.org/10.1080/24733938.2021.1883195> | Non-adolescent |
| 48 | Sanchez-Benito, J. L., & Izard, P. L. (2009). Food habits of sport young men need to be improved. *Revista Espanola De Nutricion Comunitaria-Spanish Journal of Community Nutrition*, *15*(2), 81-88. | Non-English |
| 49 | Sedek, R., Mohamad, M. M., & Kasim, Z. M. (2015). Knowledge, attitudes and practices on hydration and fluid replacement among endurance sports athletes in National University of Malaysia (UKM). *Pakistan Journal of Nutrition*, *14*(10), 658-665. <https://doi.org/10.3923/pjn.2015.658.665> | Non-adolescent |
| 50 | Sedek, R., & Yih, T. Y. (2014). Dietary habits and nutrition knowledge among athletes and non-athletes in National University of Malaysia (UKM). *Pakistan Journal of Nutrition*, *13*(12), 752-759. <https://doi.org/10.3923/pjn.2014.752.759> | Non-adolescent |
| 51 | Sekulic, D., Tahiraj, E., Zvan, M., Zenic, N., Uljevic, O., & Lesnik, B. (2016). Doping Attitudes and Covariates of Potential Doping Behaviour in High-Level Team-Sport Athletes; Gender Specific Analysis. *Journal of Sports Science & Medicine*, *15*(4), 606-615. | Non-adolescent |
| 52 | Shriver, L. H., Betts, N. M., & Wollenberg, G. (2013). Dietary intakes and eating habits of college athletes: are female college athletes following the current sports nutrition standards? *Journal of American College Health*, *61*(1), 10-16. [https://dx.doi.org/10.1080/07448481.2012.747526](https://doi.org/https://dx.doi.org/10.1080/07448481.2012.747526) | Non-adolescent |
| 53 | Soo, K., & Naughton, G. (2007). The hydration profile of female cricket players during competition. *International Journal of Sport Nutrition & Exercise Metabolism*, *17*(1), 14-26. <https://doi.org/10.1123/ijsnem.17.1.14> | Non-adolescent |
| 54 | Spronk, I., Heaney, S. E., Prvan, T., & O'Connor, H. T. (2015). Relationship Between General Nutrition Knowledge and Dietary Quality in Elite Athletes. *International Journal of Sport Nutrition & Exercise Metabolism*, *25*(3), 243-251. <https://doi.org/10.1123/ijsnem.2014-0034> | Duplicate data reported in another article |
| 55 | Tam, R., Beck, K. L., Manore, M. M., Gifford, J., Flood, V. M., & O'Connor, H. (2019). Effectiveness of Education Interventions Designed to Improve Nutrition Knowledge in Athletes: A Systematic Review. *Sports Medicine*, *49*(11), 1769-1786. <https://doi.org/10.1007/s40279-019-01157-y> | Wrong study design (systematic literature review) |
| 56 | Tawfik, S., El Koofy, N., & Moawad, E. M. I. (2016). Patterns of nutrition and dietary supplements use in young Egyptian athletes: A community-based cross-sectional survey. *PLoS ONE*, *11*(8). <https://doi.org/10.1371/journal.pone.0161252> | Mean NK score not reported |
| 57 | Torres-McGehee, T. M., Pritchett, K. L., Zippel, D., Minton, D. M., Cellamare, A., & Sibilia, M. (2012). Sports Nutrition Knowledge Among Collegiate Athletes, Coaches, Athletic Trainers, and Strength and Conditioning Specialists. *Journal of Athletic Training (National Athletic Trainers' Association)*, *47*(2), 205-211. <https://doi.org/10.4085/1062-6050-47.2.205> | Non-adolescent |
| 58 | Trabucco, G., Nikolić, M., & Vuković-Mirković, B. (2013). Nutritional knowledge and behavior among students practicing sports: Comparison between two countries. *Acta Facultatis Medicae Naissensis*, *30*(4), 201-208. <https://doi.org/10.2478/afmnai-2013-0011> | Non-adolescent |
| 59 | Trakman, G. L., Forsyth, A., Hoye, R., & Belski, R. (2019). Australian team sports athletes prefer dietitians, the internet and nutritionists for sports nutrition information. *Nutrition & Dietetics*, *76*(4), 428-437. <https://doi.org/10.1111/1747-0080.12569> | Non-adolescent |
| 60 | Trakman, G. L., Forsyth, A., Middleton, K., Hoye, R., Jenner, S., Keenan, S., & Belski, R. (2018). Australian Football Athletes Lack Awareness of Current Sport Nutrition Guidelines. *International Journal of Sport Nutrition & Exercise Metabolism*, *28*(6), 644-650. [https://dx.doi.org/10.1123/ijsnem.2018-0002](https://doi.org/https://dx.doi.org/10.1123/ijsnem.2018-0002) | Non-adolescent |
| 61 | Turner, O., Mitchell, N., Ruddock, A., Purvis, A., & Ranchordas, M. (2021). Elite squash players nutrition knowledge and influencing factors. *Journal of the International Society of Sports Nutrition*, *18*(1). <https://doi.org/10.1186/s12970-021-00443-3> | Non-adolescent |
| 62 | Tutkun, E. (2020). An Examination of Nutritional Approaches and Stress. *Progress in Nutrition*, *22*(3). <https://doi.org/10.23751/pn.v22i3.8409> | Non-adolescent |
| 63 | Updegrove, N. A., & Achterberg, C. L. (1991). The conceptual relationship between training and eating in high school distance runners. *Journal of Nutrition Education*, *23*(1), 18-24. <https://doi.org/10.1016/S0022-3182(12)80450-X> | Non-English |
| 64 | Valliant, M. W., Pittman Emplaincourt, H., Kieckhaefer Wenzel, R., & Hilson Garner, B. (2012). Nutrition Education by a Registered Dietitian Improves Dietary Intake and Nutrition Knowledge of a NCAA Female Volleyball Team. *Nutrients*, *4*(6), 506-516. <https://doi.org/10.3390/nu4060506> | Non-adolescent |
| 65 | Voser, R. D., Cunha, G. D., Marques, P. A., Duarte, M., Aimi, G. A., & Hein, A. P. (2018). Nutritional knowledge, food profile and nutritional status of futsal university players. *Rbne-Revista Brasileira De Nutricao Esportiva*, *12*(71), 394-403. | Non-English |
| 66 | Walter, O., Bobrov, A., & Tamir, S. (2018). Surprising Advantages of Low Self-Efficacy Revealed in a Sports Nutrition Education. *American Journal of Health Behavior*, *42*(4), 23-33. <https://doi.org/10.5993/AJHB.42.4.3> | No quantitative NK score |
| 67 | Whitehouse, G., & Lawlis, T. (2017). Protein supplements and adolescent athletes: A pilot study investigating the risk knowledge, motivations and prevalence of use. *Nutrition & Dietetics*, *74*(5), 509-515. <https://doi.org/10.1111/1747-0080.12367> | Non-adolescent |
| 68 | Wilson, P. B., Madrigal, L. A., & Burnfield, J. M. (2016). Weight control practices of Division I National Collegiate Athletic Association athletes. *Physician & Sportsmedicine*, *44*(2), 170-176. [https://doi.org/10.1080/00913847.2016.1149420](https://doi.org/https://dx.doi.org/10.1080/00913847.2016.1149420) | Non-adolescent |
| 69 | Yannakoulia, M., Sitara, M., & Matalas, A. L. (2002). Reported eating behavior and attitudes improvement after a nutrition intervention program in a group of young female dancers. *International Journal of Sport Nutrition and Exercise Metabolism*, *12*(1), 24-32. <https://doi.org/10.1123/ijsnem.12.1.24> | Non-adolescent |
| 70 | Yeargin, S. W., Casa, D. J., Judelson, D. A., McDermott, B. P., Ganio, M. S., Lee, E. C., Lopez, R. M., Stearns, R. L., Anderson, J. M., Armstrong, L. E., Kraemer, W. J., & Maresh, C. M. (2010). Thermoregulatory responses and hydration practices in heat-acclimatized adolescents during preseason high school football. *Journal of Athletic Training (National Athletic Trainers' Association)*, *45*(2), 136-146. <https://doi.org/10.4085/1062-6050-45.2.136> | Mean NK score not reported |
| 71 | Zawila, L. G., Steib, C. M., & Hoogenboom, B. (2003). The female collegiate cross-country runner: nutritional knowledge and attitudes. *Journal of Athletic Training (National Athletic Trainers' Association)*, *38*(1), 67-74. | Non-adolescent |
| 72 | Zeng, D., Fang, Z.-L., Qin, L., Yu, A.-Q., Ren, Y.-B., Xue, B.-Y., Zhou, X., Gao, Z.-Y., Ding, M., An, N., & Wang, Q.-R. (2020). Evaluation for the effects of nutritional education on Chinese elite male young soccer players: The application of adjusted dietary balance index (DBI). *Journal of Exercise Science & Fitness*, *18*(1), 1-6. <https://doi.org/10.1016/j.jesf.2019.08.004> | No quantitative NK score |
| 73 | Zeng, Z. H., Meng, W. Y., Sun, P., & Xie, L. S. (2019). An Exploratory Study of Youth Soccer Players' Participation Motivation and Health-Related Behaviors. *Physical Educator-Us*, *76*(2), 329-356. <https://doi.org/10.18666/tpe-2019-v76-i2-8499> | No quantitative NK score |
| 74 | Zuniga, K. E., Downey, D. L., McCluskey, R., & Rivers, C. (2017). Need for and Interest in a Sports Nutrition Mobile Device Application Among Division I Collegiate Athletes. *International Journal of Sport Nutrition & Exercise Metabolism*, *27*(1), 43-49. <https://doi.org/10.1123/ijsnem.2015-0305> | Mean NK score not reported |
